# Properties of Novel Components of Polysaccharides Isolated From *Lycium barbarum* Fruits

**DOI:** 10.1155/ijfo/4899439

**Published:** 2025-11-28

**Authors:** Yuling Chen, Xueluan Liu, Genglin Lu, Xuan He, Dandan Li, Yunong Tian, Xiaolin Feng, Yong Ye

**Affiliations:** ^1^ Department of Pharmaceutical Engineering, School of Chemistry and Chemical Engineering, South China University of Technology, Guangzhou, China, scut.edu.cn; ^2^ Department of R&D, Ganzhou Hake Biotech Co., Ltd, Ganzhou, China; ^3^ Department of Forestry, Jiangxi Environmental Engineering Vocational College, Ganzhou, China

**Keywords:** antioxidant, healthcare, isolation, *Lycium barbarum* polysaccharides, tyrosinase

## Abstract

This study has established structure‐function relationships of *Lycium barbarum* polysaccharides (LBPs) relevant to antioxidant and tyrosinase‐inhibitory functions in food systems by integrating optimized extraction, stepwise purification, comprehensive structural analysis, and functional verification, offering a novel multimethod approach to explore structure‐based mechanisms of bioactivity. Firstly, hot‐water extraction optimized at 30 mL/g, 90°C, and 5 h yielded 7.44*%* ± 0.17*%* crude LBPs. Secondly, ion‐exchange followed by gel‐filtration chromatography separated two purified fractions: LBP‐1 (115.1 kDa) and LBP‐2 (73.5 kDa). LBP‐1 exhibited a compact morphology and featured spectral characteristics indicative of a triple‐helix structure, along with both *α*‐ and *β*‐glycosidic linkages. In contrast, LBP‐2 showed a more amorphous configuration without triple‐helix features and predominantly *β*‐linkages. Their monosaccharide profiles also differed, with LBP‐1 enriched in glucose, mannuronic acid, galacturonic acid, and glucuronic acid, while LBP‐2 contained glucose, galacturonic acid, mannuronic acid, and arabinose. Both fractions exhibited in vitro antioxidant and tyrosinase‐inhibitory activities, with LBP‐2 demonstrating relatively stronger effects. The enhanced performance of LBP‐2 may be associated with its dispersed structure and uronic acid–rich *β*‐linked motifs, which increase the accessibility of functional sites. These insights support the potential application of both LBP‐1 and LBP‐2 in functional foods, with LBP‐2 showing greater promise for use in beverages and fresh‐cut fruit products requiring natural antibrowning and antioxidant solutions.

## 1. Introduction

*Lycium barbarum* polysaccharides (LBPs) are major bioactive constituents of *Lycium barbarum* L., a medicinal and edible plant widely cultivated in the Ningxia Hui Autonomous Region of northwestern China. With broad biological activities, including antioxidant, anti‐inflammatory, and immunomodulatory effects, LBPs have attracted considerable attention in recent decades. However, their incorporation into high‐value food applications remains limited due to challenges in structural definition, purification, and functionality control [1–6]. This disconnect highlights the gap between biological potential and practical implementation.

Conventional techniques such as water–alcohol precipitation, aqueous two‐phase systems, and membrane separation have been widely used for the extraction and preliminary purification of LBPs [7–9]. Gong et al. [10] employed water–alcohol precipitation to isolate LBPs and investigated their proapoptotic effects on melanoma cells. Hu et al. [11] used an aqueous two‐phase system under mild conditions to extract LBPs and assessed their antioxidant properties. Tang et al. [12] applied molecular weight–based membrane separation to obtain LBP fractions and examined their effects on human hepatocellular carcinoma cells. Despite these advancements, limited selectivity, coelution of impurities, and potential structural degradation often result in fractions lacking clearly defined molecular features, thereby hindering their use in reproducible structure–function evaluations.

A key limitation is the lack of systematic evidence linking specific structural features of LBPs to their functional performance under food‐relevant conditions. Although bioactivity assays such as antioxidant and tyrosinase inhibition tests are widely used, few studies have investigated how differences in glycosidic linkage patterns, molecular weight distribution, or sugar acid composition contribute to functional variations. The absence of this knowledge constrains both mechanistic understanding and practical standardization and continues to hinder the transformation of LBPs from health‐promoting extracts into clean‐label, functionality‐oriented food ingredients [13–17].

To overcome these challenges, this study employed response surface methodology (RSM) to optimize hot‐water extraction, followed by ion‐exchange chromatography and gel filtration chromatography to obtain two highly purified LBP fractions with distinct structural profiles [18, 19]. These chromatographic techniques were selected due to their high selectivity and ability to preserve polysaccharide integrity during purification [20]. Structural features including monosaccharide composition, glycosidic linkage types, supramolecular morphology, and apparent molecular weight were comprehensively characterized by ultraviolet (UV) spectroscopy, Fourier‐transform infrared spectroscopy (FTIR), and nuclear magnetic resonance (NMR). Functional evaluation was carried out through standardized in vitro antioxidant and tyrosinase inhibition assays. To further elucidate the mechanistic basis of the observed bioactivity differences, dominant monosaccharides in the LBP fractions were docked to tyrosinase protein to identify potential binding modes and interaction sites. By integrating structural characterization, functional assessment, and molecular docking, this study provides a comprehensive framework for elucidating the structure–activity relationships of LBPs. The findings aim to support the development of clean‐label, antioxidant and antibrowning agents derived from LBPs, with direct relevance to applications in beverages, fresh‐cut fruits, and other minimally processed food systems.

## 2. Material and Methods

### 2.1. Materials

Dried Ningxia *Lycium barbarum* fruit was purchased from Guangdong Fengchun Pharmaceutical Company Limited; bovine serum protein (96%) and papain (>2000 U/mg) were purchased from Aladdin Biochemical Science and Technology Company Limited; and Caulmers Brilliant Blue G‐250 (AR), L‐dopa (levodopa 99%) and tyrosinase (≥ 500 U/mg) were purchased from Shanghai McLean Biochemistry and Technology Company Limited.

### 2.2. Extraction of LBPs

#### 2.2.1. Sample Pretreatment

*Lycium barbarum* fruit was washed, dried, and crushed. n‐Hexane was added at a ratio of 6 mL/g of dried fruit, and the mixture was refluxed at 70°C for 5 h. After filtration, the residue was air‐dried and then vacuum‐dried at 50°C for 12 h to ensure complete removal of residual solvent. The degreased *Lycium barbarum* was confirmed to be free of residual n‐hexane by gas chromatography with flame ionization detection (GC‐FID) using headspace sampling. No detectable residue was observed under the detection limit of 0.5 ppm.

#### 2.2.2. Single‐Factor Experiment

The LBPs were extracted from defatted *Lycium barbarum* powder using hot water [21]. The effects of extraction conditions—including a liquid‐to‐solid ratio of 15–35 mL/g, temperature of 60°C–100°C, and extraction time of 1–5 h—were systematically investigated and optimized to improve polysaccharide yield. After extraction, the suspension was centrifuged at 8000 rpm for 5 min, and the supernatant was collected. It was then concentrated to one‐fifth of its original volume by rotary evaporation at 55°C under reduced pressure. The concentrated extract was added dropwise into four volumes of prechilled 95% ethanol under gentle stirring at room temperature. The mixture was then stored at 0°C–4°C for at least 12 h to allow full polysaccharide precipitation. The resulting precipitate was collected by centrifugation at 8000 rpm for 5 min and redissolved in a small volume of deionized water.

The solution was frozen at −20°C and freeze‐dried under vacuum conditions. The cold trap temperature was maintained at 60°C, the vacuum pressure was held below 20 Pa, and the drying duration was 24 h. The entire extraction procedure was performed in triplicate.

#### 2.2.3. Determination of the Content of LBPs

Phenol‐sulfuric acid color development method was used to determine the yield of LBP [22]. The glucose (Glc) standard solutions of 10, 20, 30, 40, 50, 60, 70, and 80 *μ*g/mL were added 1.0 mL of 5% phenol solution and 5.0 mL of sulfuric acid sequentially and stood for 10 min, shaken well, and then kept at 40°C for 15 min, then cooled down to room temperature, and the deionized water was used as a blank control to determine the absorbance value of each Glc standard solution at 490 nm. The Glc standard curve (*y* = 10.769*x* + 0.131, *R* ^2^ = 0.9987) was plotted. The crude extract of LBPs was dissolved in deionized water and diluted to the appropriate concentration and added the reagents in the same way as the standard, and the absorbance at 490 nm was measured, and the content of polysaccharide was calculated according to the standard curve equation, and calculated according to Formula (1), and three parallel experiments were carried out for each sample.

(1) LBP%=c×Vm×103×100%,

where *c* is the concentration of extract (mg/mL), *V* is the total volume of samples (mL), and *m* is the dry weight of *Lycium barbarum* (g).

#### 2.2.4. Response Surface Optimization of the Extraction Process

Based on single‐factor experiments, the liquid/solid ratio (*A*), extraction temperature (*B*), and extraction duration (*C*) were taken as the independent variable factors, and the response surface optimization experiments were carried out by using Design‐Expert v10.0.4 software with the yield of LBP as the index and the design results of the response surface experiments were shown in Table 1.

**Table 1:** Response surface test design factors.

**Level**	**Factor**		
	**Liquid/feed ratio (mL/g)**	**Extraction temperature (°C)**	**Extraction duration (h)**
−1	20	70	3
0	25	80	4
1	30	90	5

### 2.3. Isolation of Subfractions From the Crude LBPs

#### 2.3.1. Protein Removal and Quantification

Papain‐assisted enzymatic digestion and Sevage treatment were used to remove protein residues [23]. Briefly, 0.1 g of crude LBPs and 0.1 g of papain (from Carica papaya, Sigma‐Aldrich, ≥ 95% purity, ≥ 30 U/mg activity) were dissolved in 100 mL of deionized water and incubated at 60°C, pH 7.0 for 1 h. The mixture was centrifuged at 8000 r/min for 5 min to collect the supernatant. An organic extraction solution (chloroform: n‐butanol = 5 : 1, *v*/*v*) at four times the sample volume was then added, followed by vigorous shaking for 20 min. After centrifugation under the same conditions, the upper aqueous phase was retained. The Sevage step was repeated until no visible protein layer remained. The final supernatant was freeze‐dried to obtain deproteinized LBPs. All samples were stored in airtight containers at 4°C prior to further analysis.

Protein content before and after treatment was quantified using the bicinchoninic acid (BCA) assay, with BSA as the standard. Absorbance was recorded at 595 nm using a microplate reader, and protein concentration was calculated from the BSA calibration curve. The protein removal rate (%) was calculated using the following Equation (2):

(2) Protein removal rate%=C0−C1C0×100%,

where *C*_0_ and *C*_1_ represent the protein concentrations (mg/mL) before and after treatment, respectively.

SDS‐PAGE was conducted to further verify protein removal. Samples before and after deproteinization were dissolved in deionized water, mixed with loading buffer, and boiled for 5 min. Electrophoresis was performed on a 12% polyacrylamide gel at 120 V for approximately 90 min. Gels were stained with Coomassie Brilliant Blue R‐250 and destained with a methanol–acetic acid solution. The disappearance of visible protein bands after treatment was used as qualitative evidence of effective protein elimination.

#### 2.3.2. Purification of LBPs

The deproteinized LBP solution was first subjected to DEAE‐52 cellulose column chromatography for further purification [24]. DEAE‐52 cellulose was pretreated by soaking sequentially in 0.5 mol/L sodium hydroxide, hydrochloric acid, and sodium hydroxide for 2 h each, followed by thorough rinsing with deionized water until neutral. The treated resin was then stored in deionized water.

The pretreated DEAE‐52 cellulose was wet‐packed into a column (*ϕ* 2.5 × 60 cm), and 10 mL of the LBP solution (20 mg/mL) was loaded. The column was eluted stepwise with deionized water, 0.1 mol/L NaCl, 0.3 mol/L NaCl, and 0.5 mol/L NaCl, at a flow rate of 1.0 mL/min. Eluates were collected in 5‐mL fractions, and the carbohydrate content was measured by the phenol‐sulfuric acid method at 490 nm. Fractions corresponding to similar absorbance peaks were combined and concentrated under reduced pressure at 50°C and −0.08 MPa, followed by freeze‐drying at −60°C under 10 Pa for 24 h.

The freeze‐dried eluates were redissolved in a small volume of deionized water and applied to a Sephadex G‐150 column (*ϕ* 2.6 × 100 cm) that had been preswollen in deionized water for 24 h [25]. The column was equilibrated with deionized water and eluted at a constant flow rate of 0.5 mL/min. Eluates were collected in 5‐mL fractions, and carbohydrate content was determined as described above. Fractions with similar absorbance profiles were pooled, concentrated under reduced pressure at 50°C and −0.08 MPa, and finally freeze‐dried under 10 Pa at −60°C for 24 h to obtain the purified LBP subfractions.

### 2.4. Structural Characterization of Subfractions of LBP

#### 2.4.1. UV‐vis and IR Spectral Determination

UV‐vis and IR spectral analyses were carried out on the purified LBP subfractions LBP‐1 and LBP‐2 and the crude LBPs. Deionized water was used as blank, and UV‐2450 UV‐visible spectrophotometer (Shimadzu Corporation, Kyoto, Japan) was used for scanning in the wavelength range of 220~600 nm. The infrared spectra were scanned and recorded in the range of 500–4000 cm^−1^ by KBr pressing method using FTIR VERTEX 70 (Bruker Corporation, Bremen, Germany).

#### 2.4.2. NMR Spectrum Analysis

Then, 10 mg of LBP‐1 and LBP‐2 were dissolved in 0.6 mL of D_2_O, and the ^1^H NMR spectra were determined using a NMR instrument (AVANCE III HD500) (Bruker Corporation, Bremen, Germany), at 25°C.

#### 2.4.3. Molecular Weight Measurement

The experiment was performed using Thermo’s Ultimate 3000 high‐performance liquid chromatography with a RefractoMax 521 oscillometric detector (Thermo Fisher Scientific, Waltham, MA, United States). The chromatographic conditions were Ultrahydrogel 1000 column (7.8 × 300 mm) and Ultrahydrogel 500 column (7.8 × 300 mm) in tandem, flow phase 20 mmol/L KH_2_PO_4_ buffer solution, flow rate 0.8 mL/min, injection volume 20 *μ*L, run time 35 min, and column temperature 35°C.

A series of dextran standard solutions (2 mg/mL) with different molecular weights (Mw: 5200, 11,600, 23,800, 48,600, 148,000, 273,000, 410,000, and 668,000 Da) were prepared in 20 mmol/L KH_2_PO_4_ solution, and the retention time was detected by HPLC. The standard curve of molecular weight versus the retention time was plotted. Under the same chromatographic conditions, the samples of LBP‐1 and LBP‐2 with the concentration of 2 mg/mL were injected to determine the retention time, and their molecular weight was calculated according to the standard curve.

#### 2.4.4. Observation by Scanning Electron Microscope

LBP‐1 and LBP‐2 samples were evenly mounted onto conductive adhesive tapes, sputter‐coated with a gold layer of approximately 10 nm, and examined using a Phenom ProX scanning electron microscope (Phenom‐World B.V., Eindhoven, Netherlands) at an accelerating voltage of 10 kV and a working distance of 10 mm.

#### 2.4.5. X‐Ray Diffraction (XRD) Detection

A D8 Advanced X‐ray diffractometer (Bruker Corporation, Bremen, Germany) was used to analyze the crystallinity of LBP‐1 and LBP‐2. Cu‐K*α* was used as the radiation source, with a working voltage of 40 kV and current of 40 mA. The scanning range was 2*θ* = 5 ~ 90^°^, using a continuous scanning mode at a speed of 5°/min. Crystallinity was estimated by the peak area method, based on the ratio of crystalline to total (crystalline + amorphous) peak areas obtained through deconvolution of the XRD profiles.

#### 2.4.6. Detection of Triple‐Stranded Helical Structure

Congo red was a dye with the molecular formula C_32_H_22_N_6_S_2_Na_2_. In alkaline solution, Congo red could form complexes with polysaccharides containing triple‐stranded helical structures. In a very low concentration of NaOH solution, the maximum absorption wavelength of the complex would be significantly red‐shifted compared with the Congo red blank sample, and while increasing the concentration of NaOH solution, the maximum absorption wavelength of the complex would be reduced [26]. The procedure was as follows: 2 mL of 2 mg/mL LBP‐1 and LBP‐2 solution and 2 mL of 80 *μ*mol/L Congo red solution were mixed, and then, 1 mol/L NaOH solution was added to the respective mixture so that the final concentration of NaOH solution was controlled within the range of 0–0.5 mol/L, and the difference between two concentrations was 0.05 mol/L. The reaction was carried out at room temperature for 10 min and then scanned in the wavelength range of 400–600 nm to determine the maximum absorption wavelengths of the different concentrations, with deionized water replacing the polysaccharide samples as the blank control, and kombucha polysaccharide and gel polysaccharide as the positive control. The maximum absorption wavelengths measured for each concentration were used as vertical coordinates, and the NaOH concentration in the corresponding mixtures was used as horizontal coordinates to plot the experimental results.

#### 2.4.7. Detection of Monosaccharides

Monosaccharide composition was determined using ion chromatography (IC) equipped with a Dionex ICS‐5000+ system (Thermo Fisher Scientific, United States), using a CarboPac PA20 column (3 × 150 mm) and AERS 500 suppressor. Elution was performed with 10 mM NaOH as the mobile phase at a flow rate of 0.5 mL/min. The column temperature was maintained at 30°C, and the injection volume was 10 *μ*L. Detection was achieved via pulsed amperometric detection (PAD).

The standard monosaccharides Fuc, rhamnose (Rha), arabinose (Ara), galactose (Gal), Glc, xylose (Xyl), mannose (Man), Fru, Rib, galacturonic acid (Gal‐UA), guluronic acid (Gul‐UA), glucuronic acid (Glc‐UA), and mannuronic acid (Man‐UA) were dissolved to make a standard solution of 10 mg/mL with deionized water. The standard solutions were then prepared into standard mixing solutions with concentrations of 60, 50, and 40 *μ*g/mL, respectively. Fuc, Rha, Ara, Gal, Glc, and Xyl were mixed according to the concentration gradient of 40, 32, 24, 16, 8, 4, 0.8, and 0.4 *μ*g/mL; Man was prepared to the concentration gradient of 50, 40, 30, 20, 10, 5, 1.0, and 0.5 *μ*g/mL; and Fru, Rib, Gal‐UA, Gul‐UA, Glc‐UA, Fru, Rib, Gal‐UA, Gul‐UA, Glc‐UA, and Man‐UA were prepared into standards of 60, 48, 36, 24, 12, 6, 1.2, and 0.6 *μ*g/mL, and each concentration gradient of the standards was detected by IC, and the peak area was recorded and plotted as the vertical coordinates, and the concentration of the standards was plotted as the horizontal coordinates to draw the standard curve.

Then, 5 mg of LBP‐1 and LBP‐2 was added to 1 mL of 2 mol/L trifluoroacetic acid (TFA) and heated at 120°C for 2 h. TFA was removed by nitrogen blow‐drying. The residue was washed with 1 mL methanol and dried, repeated 2–3 times. The resulting sample was dissolved in deionized water and filtered through a 0.22‐*μ*m membrane prior to injection. Monosaccharide content was calculated from the standard curve based on the peak area of each corresponding monosaccharide [27].

### 2.5. Differential Scanning Calorimetry (DSC) Analysis

To evaluate the thermal transition characteristics and molecular organization of different LBP subfractions, DSC was performed. Thermal parameters such as glass transition temperature (*T*_g_) can provide insights into chain mobility, thermal stability, and the presence of ordered conformations such as triple‐helical structures, which are relevant to the biofunctionality of polysaccharides.

Approximately 10 mg of freeze‐dried LBP‐1 or LBP‐2 was sealed in an aluminum crucible and scanned from 30°C to 200°C at a heating rate of 10°C/min under a nitrogen flow of 50 mL/min. An empty aluminum pan served as the reference.

### 2.6. Determination of Antioxidant Activity

LBP, LBP‐1, and LBP‐2 solutions with concentrations of 0.5, 1.0, 2.5, 5.0, 10, and 20 mg/mL were prepared with deionized water.

#### 2.6.1. ABTS^+^ Radical Scavenging Capacity

ABTS^+^ radical scavenging capacity was determined referring to the method of Jhan [28]. The 7.5 mmol/L ABTS^+^ reserve solution was prepared with 2.6 mmol/L potassium persulfate methanol solution and stored in a dry place away from light overnight. Then, 1 mL of the reserve solution was taken up and diluted with methanol, so that the absorbance value of the solution at 744 nm was measured near 0.710 ± 0.020, and the ABTS^+^ solution was obtained. Then, 150 *μ*L of each concentration of LBP‐1, LBP‐2, and crude LBP solution were added, mixed uniformly, and then placed in a dark place away from the light for 2 h. The absorbance values (*A*) of each concentration were tested at 744 nm. VC and arbutin were used as the control, the experiments were repeated three times, and the ABTS^+^ radical scavenging rate was calculated according to Equation (3).

(3) Radical scavenging rate %=1−Asample−AcontrolAblank×100%.

#### 2.6.2. DPPH Radical Scavenging Capacity

DPPH radical scavenging was determined by the method of Chen et al. [29]. DPPH‐solution was prepared with 95% ethanol at a concentration of 0.2 mmol/L. Then, 2 mL of each concentration of LBP‐1, LBP‐2, and crude LBP solution was added to 2.0 mL of DPPH–ethanol solution, mixed homogeneously, and then kept dry and protected from light at room temperature for 30 min. Another 2.0 mL of distilled water and 2.0 mL of 95% ethanol were mixed homogeneously to do the zero adjustment. The absorbance values (*A*) of each concentration mixture were tested at 517 nm and recorded. VC and arbutin were used as the control, and the experiments were repeated three times to calculate the DPPH radical scavenging rate according to Formula (3).

#### 2.6.3. Hydroxyl Radical Scavenging Capacity

Then, 0.2 mL of each concentration of LBP‐1, LBP‐2, and crude LBP solution was taken and mixed with 1.0 mL of 2.0 mmol/L FeSO_4_ solution, 0.4 mL of 2.0 mmol/L sodium salicylate solution, and 1.0 mL of 0.1% H_2_O_2_ solution. The solution was placed in 37°C for 1 h and then cooled to room temperature, and the absorbance value (*A*) was measured at 510 nm [30]. VC and arbutin were used as controls, and the experiments were repeated three times. The hydroxyl radical scavenging rate was calculated according to Equation (3).

### 2.7. The Inhibition of Tyrosinase Activity

#### 2.7.1. Determination of Tyrosinase Inhibition Activity

Then, 1 mL of LBP‐1 and LBP‐2 solutions with 0, 5, 10, and 15 mg/mL was taken, respectively, and 7.0 mL of PBS buffer solution (pH = 6.8), 1.0 mL of L‐dopa solution (10 mmol/L), and 1.0 mL of aqueous tyrosinase solution (10 mg/L) were added, and the reaction was carried out at 37°C, and the change of absorbance value (*A*) of the mixture at 475 nm in the reaction time of 0–20 min was recorded [31] with deionized water instead of polysaccharide sample as a blank group and deionized water instead of reagent as a background group. The tyrosinase inhibition was calculated according to Equation (4). The experiment was repeated three times.

(4) Tyrosinase inhibition%=1−Asample−AbackgroundAblank×100%.

#### 2.7.2. Kinetic Analysis of Tyrosinase Activity Inhibition

The initial rate of the reaction was determined by fixing the concentration of tyrosinase at 1.0 mg/L and changing the concentration of L‐dopa to 0.25, 0.5, 1, and 2 mmol/L, and the concentrations of LBP‐1 and LBP‐2 were 0, 0.5, 1.5, and 2.0 mg/mL. The kinetic curves of the inhibition of tyrosinase activity by LBP‐1 and LBP‐2 were plotted according to the Lineweaver–Burk double inverse plots of enzyme reaction rate and substrate (L‐dopa) concentration.

### 2.8. Molecular Docking Simulation

To investigate the potential interactions between tyrosinase and key monosaccharides present in the LBP fractions, molecular docking simulations were performed using AutoDock Vina (Version 1.2.0). The three‐dimensional structure of mushroom tyrosinase was retrieved from the Protein Data Bank (PDB ID: 2Y9X). All crystallographic water molecules and native ligands were removed, and polar hydrogens were added using AutoDockTools (Version 1.5.7). The docking grid was defined to fully encompass the active site region, including the copper‐binding catalytic center.

Four monosaccharides were selected as ligands, namely, Ara, GalUA, Glc, and Rha, based on their differential abundance in LBP‐1 and LBP‐2. The ligand structures were obtained from the PubChem database and energy‐minimized using the MMFF94 force field in Chem3D. All ligands were then converted into PDBQT format and docked individually against the tyrosinase target. Binding affinities (expressed in kcal/mol) and docking poses were evaluated based on the predicted scores generated by AutoDock Vina. Visualization and analysis of ligand–protein interactions were conducted using PyMOL, focusing on hydrogen bonds, salt bridge formation, and potential coordination with copper ions within the active site.

### 2.9. Statistical Analyses

All experiments were conducted in triplicate, and results are presented as mean ± standard deviation (SD). Statistical analysis was performed using OriginPro 2022 (OriginLab Corporation, MA, United States). Prior to one‐way analysis of variance (ANOVA), the Shapiro–Wilk test was used to assess normality and Levene’s test was applied to evaluate homogeneity of variances. One‐way ANOVA was conducted at a significance level of *p* < 0.05, and Tukey’s post hoc test was employed for pairwise comparisons where applicable.

## 3. Results and Discussion

### 3.1. Optimization of Extraction Conditions for LBP

#### 3.1.1. Single‐Factor Experiments

To investigate the impact of the liquid versus solid ratio, extraction temperature and extraction time on the yield of crude LBP, single‐factor optimization experiments were performed with the results presented in Figure 1. In the initial series of experiments (Figure 1a), the extraction temperature and time were maintained at 80°C and 3 h, respectively, while the liquid versus solid ratio was varied from 15 to 35 mL/g. The yield increased with the liquid versus solid ratio, reaching a maximum at 30 mL/g, after which it declined, possibly due to dilution effects or decreased solvent efficiency. Consequently, a ratio of 30 mL/g was identified as the optimal condition. In the subsequent series of experiments (Figure 1b), the liquid versus solid ratio and extraction time were maintained at 30 mL/g and 3 h, respectively, while the extraction temperature was varied from 60°C to 100°C. The yield demonstrated an increasing trend with rising temperature, achieving a peak at 90°C, followed by a gradual decline. Consequently, 90°C was identified as the optimal extraction temperature. In the final series of experiments (Figure 1c), the liquid versus solid ratio and extraction temperature were held at 30 mL/g and 90°C, respectively, while the extraction time was varied from 1 to 5 h. Yield increased with time, plateauing after 3 h. To balance yield and time efficiency, an extraction time of 3 h was selected.

Figure 1Single‐factor optimization of crude LBP yield. (a) Effect of liquid vs. solid ratio. (b) Effect of extraction temperature. (c) Effect of extraction time.

**Figure:**
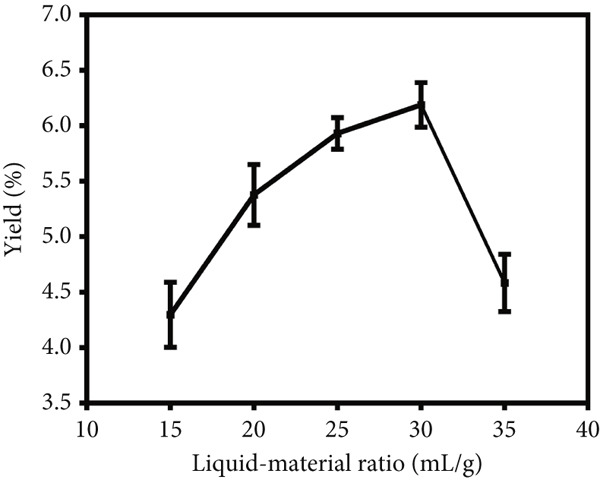
(a)

**Figure:**
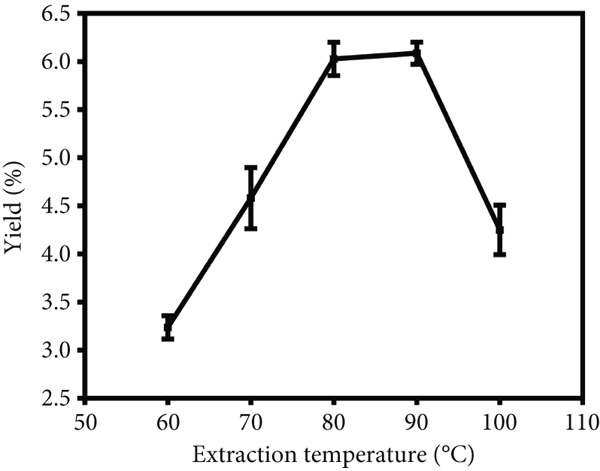
(b)

**Figure:**
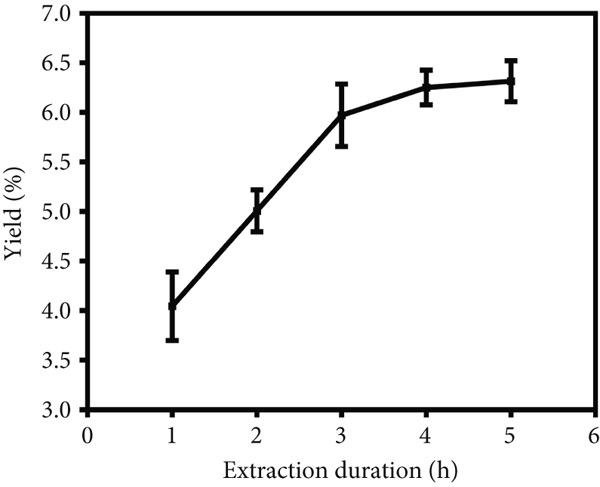
(c)

The single‐factor experiments identified preliminary optimal conditions for LBP extraction, including a liquid‐to‐solid ratio of 30 mL/g, an extraction temperature of 90°C, and an extraction time of 3 h. Under these conditions, the actual extraction yield was 7.44*%* ± 0.17*%* (*n* = 3), providing a solid empirical foundation for subsequent optimization using RSM.

#### 3.1.2. Extraction Process Optimization Using RSM

Based on the single‐factor experiment results, a Box–Behnken design was used in Design‐Expert software (Version 13.0, Stat‐Ease Inc., Minneapolis, MN, United States) to optimize the extraction process, with liquid versus solid ratio (*A*), extraction temperature (*B*), and extraction time (*C*) as independent variables. Tables 2 and 3 presented the response surface results with ANOVA, respectively. RSM produced the following regression model: *R* = 6.47 + 0.55*A* + 0.64*B* + 0.52*C* + 0.067*A* *B* + 0.028 *A* *C* − 0.038*B* *C* − 0.24*A* ^2^ − 0.25*B* ^2^ − 0.17*C* ^2^. ANOVA showed that the model was highly significant (*p* < 0.001) with a nonsignificant lack of fit (*p* = 0.1055 > 0.1), indicating a good fit. Predicted values closely matched experimental data, confirming the model′s reliability. Figure 2 illustrated the effects of liquid versus solid ratio, extraction temperature, and extraction time on polysaccharide yield. Extraction temperature (*B*) had the greatest influence on yield, followed by liquid versus solid ratio (*A*) and extraction time (*C*). Interaction effects between variables were not statistically significant (*p* > 0.05), indicating that individual factors had a stronger impact on yield than their interactions. The RSM optimal extraction conditions were a liquid versus solid ratio of 30 mL/g, an extraction temperature of 90°C, and an extraction time of 3 h. Under these optimized conditions, the RSM model predicted a polysaccharide yield of 7.56*%* ± 0.14*%*. To validate this prediction, three independent experiments were performed, yielding an actual average extraction rate of 7.44*%* ± 0.17*%* (*n* = 3). The close agreement between predicted and experimental values confirms the model′s reliability and practical applicability.

**Table 2:** Response surface test design and results.

**No.**	**A**	**B**	**C**	**Yield (%)**
1	0	1	1	7.31
2	0	−1	−1	4.72
3	−1	−1	0	4.96
4	0	1	−1	6.24
5	−1	1	0	5.92
6	1	1	0	7.15
7	−1	0	1	5.94
8	0	0	0	6.57
9	1	0	−1	6.13
10	0	−1	1	5.94
11	0	0	0	6.36
12	0	0	0	6.52
13	1	0	1	7.11
14	1	−1	0	5.92
15	0	0	0	6.48
16	0	0	0	6.41
17	−1	0	−1	5.07

**Table 3:** Analysis of variance of response surface results.

**Source**	**Sum of squares**	**Degrees of freedom**	**Mean square**	**F**	**p**	**Significance**
Model	8.51	9	0.95	58.13	< 0.0001	Significant
*A*	2.44	1	0.015	150.20	< 0.0001	
*B*	3.23	1	0.14	198.20	< 0.0001	
*C*	2.14	1	1.22	131.63	< 0.0001	
*A* *B*	0.018	1	0.018	1.12	0.3251	
*A* *C*	3.025 × 10^−3^	1	3.025 × 10^-3^	0.19	0.6793	
*B* *C*	5.625 × 10^−3^	1	5.625 × 10^−3^	0.35	0.5751	
*A* ^2^	0.23	1	0.23	14.32	0.0069	
*B* ^2^	0.25	1	0.25	15.56	0.0056	
*C* ^2^	0.12	1	0.12	7.50	0.029	
Residual	0.11	7	0.016			
Lack of fit	0.086	3	0.029	4.04	0.1055	Not significant
Pure error	0.028	4	7.07 × 10^−3^			
Cor total	8.63	16				

Figure 2Response surface diagram of the interaction between factors.

**Figure:**
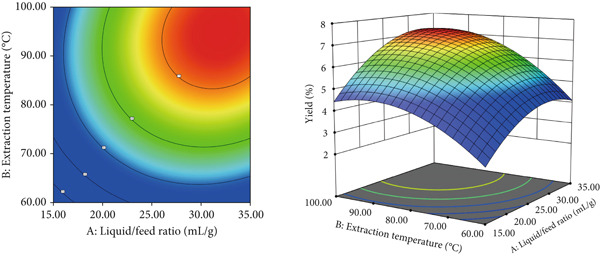
(a)

**Figure:**
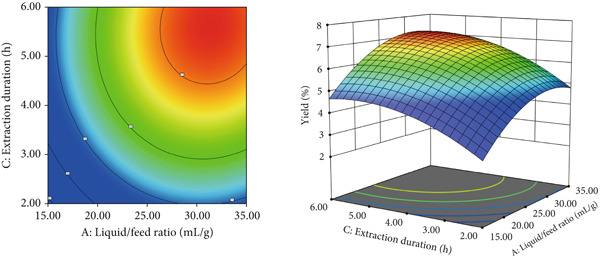
(b)

**Figure:**
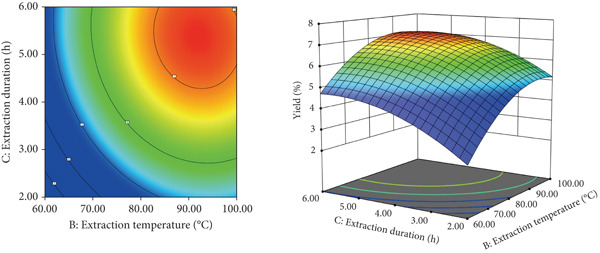
(c)

### 3.2. Protein Removal Efficiency

The combined enzymatic and Sevage treatment significantly reduced protein content in the crude LBP extract. As shown in Figure 3a, the absorbance at 595 nm, measured using the BCA assay, decreased progressively with increasing rounds of Sevage treatment. A marked decline occurred during the first two rounds, and the curve gradually stabilized in subsequent cycles, eventually reaching an absorbance of approximately 0.12. This trend suggests that most protein was removed during the early stages of the process.

Figure 3(a) Effect of Sevag and enzyme‐assisted deproteinization on polysaccharide yield. (b) Elution profile of crude LBP on a DEAE‐52 cellulose column. (c) Further separation using a Sephadex G‐150 column, yielding four purified fractions: LBP‐1, LBP‐2, LBP‐3, and LBP‐4.

**Figure:**
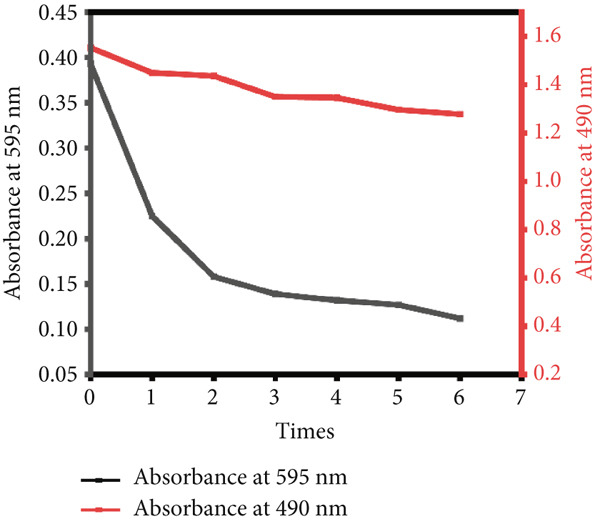
(a)

**Figure:**
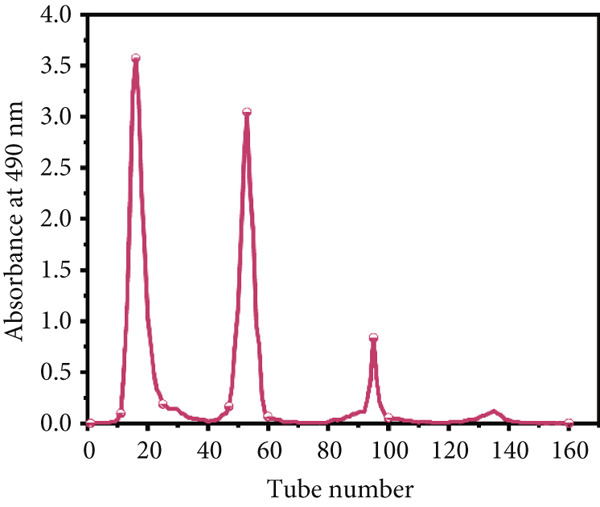
(b)

**Figure:**
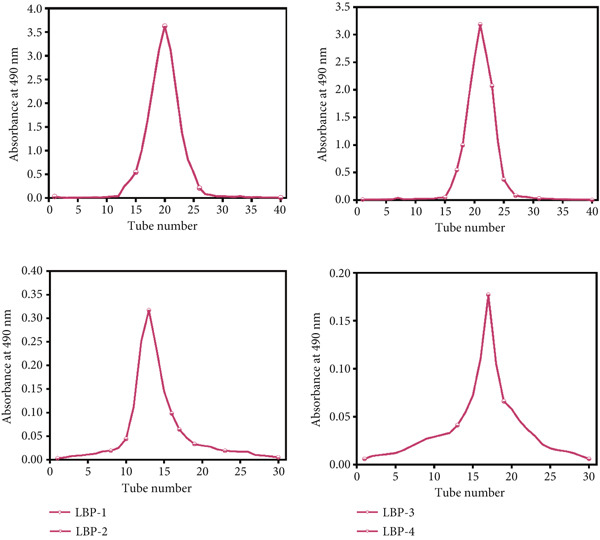
(c)

Meanwhile, the absorbance at 490 nm, reflecting polysaccharide content based on the phenol‐sulfuric acid method, remained relatively stable throughout the treatment cycles. This indicates minimal loss of polysaccharides during deproteinization. Based on the quantitative results, the protein removal rate reached 59.8%, and the polysaccharide retention rate was 92.47%. SDS‐PAGE analysis was conducted to verify protein removal. As shown in Figure S2, no visible protein bands were observed in the purified LBP sample, confirming the effective elimination of residual proteins.

### 3.3. Separation and Purification of LBP Subfractions

The crude deproteinized LBPs were further separated and purified through a two‐step chromatographic strategy. First, DEAE‐52 cellulose column chromatography was performed with stepwise elution using deionized water and NaCl solutions at 0.1, 0.3, and 0.5 mol/L. As shown in Figure 3b, four major elution peaks were observed, corresponding to preliminary polysaccharide subfractions. These were subsequently subjected to further purification via Sephadex G‐150 gel filtration chromatography. The elution curves for each subfraction are presented in Figure 3c, confirming successful resolution into four distinct purified components, designated LBP‐1, LBP‐2, LBP‐3, and LBP‐4.

Based on the mass of freeze‐dried eluates relative to the crude LBP input, the elution recovery rates were calculated as follows: LBP‐1, 46.5*%* ± 2.3*%*; LBP‐2, 28.0*%* ± 1.7*%*; LBP‐3, 4.5*%* ± 0.4*%*; and LBP‐4, 3.0*%* ± 0.3*%* (*n* = 3). Due to the low yields of LBP‐3 and LBP‐4, subsequent characterization focused on LBP‐1 and LBP‐2. The carbohydrate contents of LBP‐1 and LBP‐2, determined by the phenol‐sulfuric acid assay using Glc as standard, were 71.1*%* ± 2.6*%* and 76.5*%* ± 1.8*%*, respectively. Correspondingly, residual protein contents measured by the BCA method were below 11.0% in both fractions, confirming high polysaccharide purity after sequential purification. These results confirm that the two main fractions obtained after sequential purification are predominantly composed of polysaccharides, with minimal protein contamination.

Taken together, the combined use of DEAE‐52 ion‐exchange and Sephadex G‐150 gel filtration enabled effective separation of structurally distinct LBP subfractions. The differences in elution behavior suggest variation in molecular weight and charge among the components, reflecting the inherent heterogeneity of plant‐derived polysaccharides [32, 33].

### 3.4. UV‐vis and FTIR Spectrum Analysis

As shown in Figure 4a, UV absorption spectra of LBP‐1 and LBP‐2 displayed no significant peaks around 280 nm, suggesting the absence of aromatic protein residues that typically absorb in this region [34]. This observation aligns with the low protein content previously measured by BCA assay, confirming effective protein removal during purification.

Figure 4Spectroscopic analysis of crude and purified LBP fractions. (a) UV absorption spectra of crude LBP, LBP‐1, and LBP‐2. (b) FTIR spectra of LBP‐1 and LBP‐2. (c) ^1^H NMR spectrum of LBP‐1. (d) ^13^C NMR spectrum of LBP‐1. (e) ^1^H NMR spectrum of LBP‐2. (f) ^13^C NMR spectrum of LBP‐2.

**Figure:**
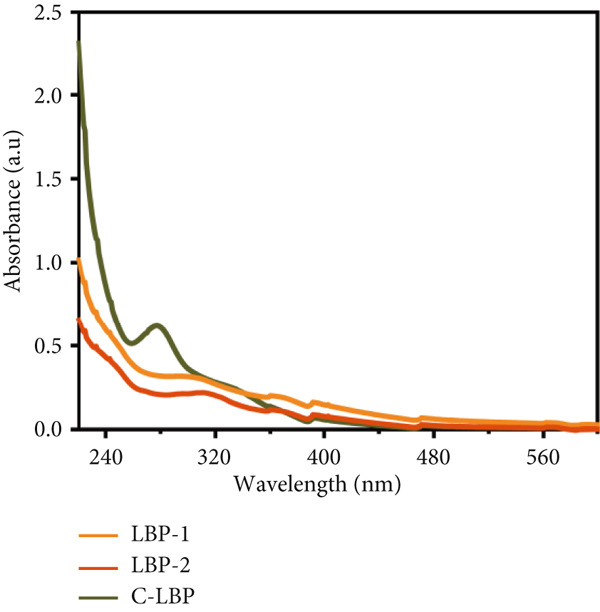
(a)

**Figure:**
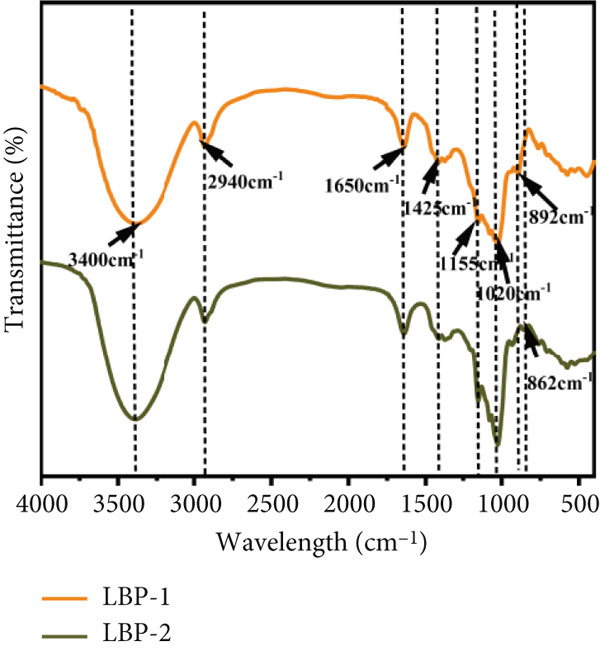
(b)

**Figure:**
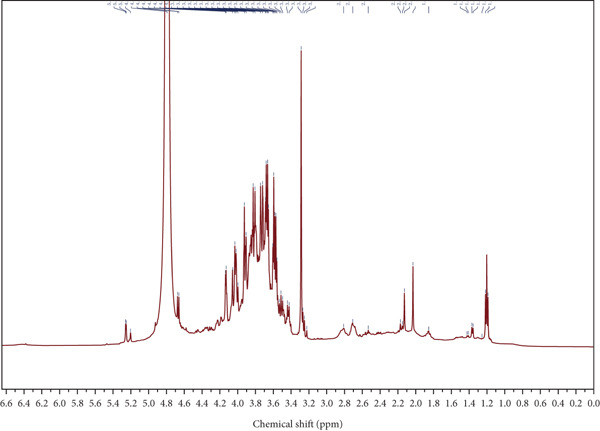
(c)

**Figure:**
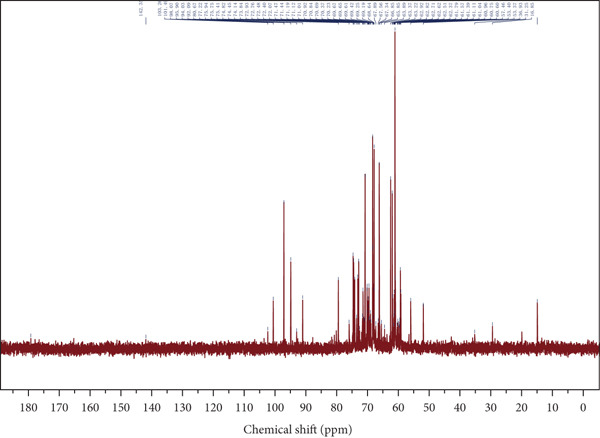
(d)

**Figure:**
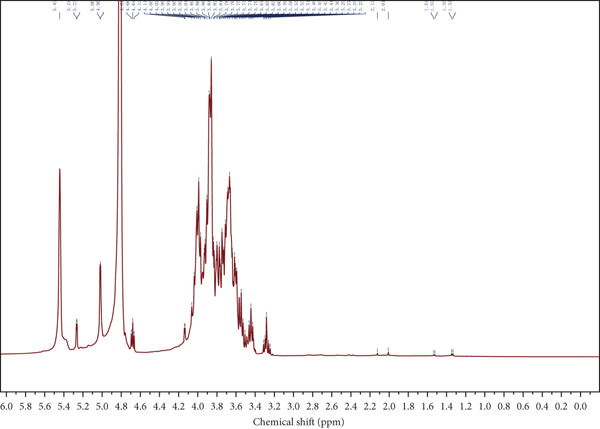
(e)

**Figure:**
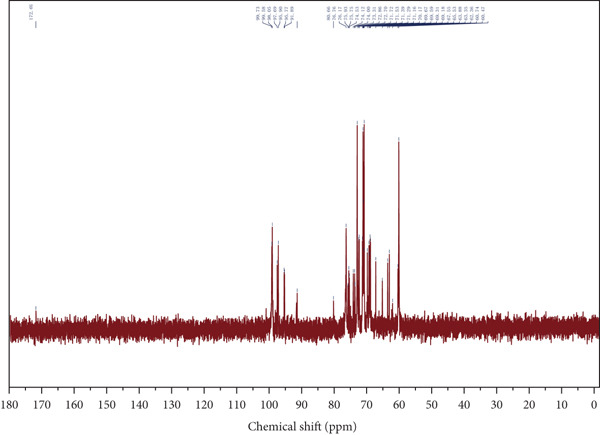
(f)

In the FTIR spectra (Figure 4b), both LBP‐1 and LBP‐2 exhibited a broad absorption band near 3400 cm^−1^, corresponding to O–H stretching vibrations, and a peak around 2940 cm^−1^ attributed to C–H stretching [35]. The strong absorption near 1650 cm^−1^ was assigned to C=O stretching, which likely reflects the presence of uronic acid residues [36]. Peaks around 1425 cm^−1^ and in the region of 1200~1020 cm^−1^ corresponded to pyranose ring deformation and C–O stretching vibrations, respectively. These C–O associated peaks were more prominent in LBP‐1, suggesting greater linkage complexity. Additionally, LBP‐1 displayed a distinct signal at 862 cm^−1^, often attributed to *α*‐anomeric glycosidic linkages, whereas LBP‐2 showed a weaker signal near 892 cm^−1^, which is typically associated with *β*‐linkages [37]. These differences in the fingerprint region suggest distinct glycosidic conformations between the two subfractions, which may underlie their differential bioactivities.

### 3.5. NMR Spectroscopy

Both ^1^H and ^13^C NMR spectra (Figures 4c, 4d, 4e, and 4f) provided detailed insights into the glycosidic bond configurations and structural features of LBP‐1 and LBP‐2. In the ^1^H NMR spectra, anomeric proton signals above *δ* 5.0 ppm are typically indicative of *α*‐glycosidic linkages, while those below *δ* 5.0 ppm suggest *β*‐configurations. LBP‐1 (Figure 4c) exhibited multiple signals in both regions, supporting the presence of mixed *α*‐ and *β*‐glycosidic bonds. In contrast, LBP‐2 (Figure 4e) showed pronounced peaks between *δ* 4.4 and approximately 4.6 ppm, consistent with a predominance of *β*‐linkages. The ^13^C NMR spectra further confirmed these structural distinctions. Anomeric carbon signals, typically found between *δ* 90 and approximately 105 ppm, were more diverse in LBP‐1, reflecting its structural heterogeneity. LBP‐2 exhibited a simpler anomeric carbon profile, in line with its more uniform backbone.

Detailed peak assignments for major sugar residues in both fractions are summarized in Table S1 (LBP‐1) and Table S2 (LBP‐2). Overall, LBP‐1 contained both *α*‐ and *β*‐glycosidic bonds with diverse backbone structures, while LBP‐2 was mainly composed of *β*‐linked monosaccharides. These structural differences may contribute to their distinct physicochemical and biological properties.

### 3.6. Molecular Weight Analysis

The molecular weights of LBP‐1 and LBP‐2 were determined using high‐performance gel permeation chromatography (HPGPC) (Figure 5a,b). LBP‐1 and LBP‐2 eluted at 20.167 and 20.975 min, respectively, each displaying single, symmetrical peaks, indicating their homogeneity. The average molecular weights were calculated as 115.1 kDa for LBP‐1 and 73.5 kDa for LBP‐2, confirming distinct molecular sizes between the two subfractions [38].

Figure 5Structural characterization of LBP‐1 and LBP‐2. (a) HPGPC chromatogram of LBP‐1. (b) HPGPC chromatogram of LBP‐2. (c, d) SEM images of LBP‐1. (e, f) SEM images of LBP‐2.

**Figure:**
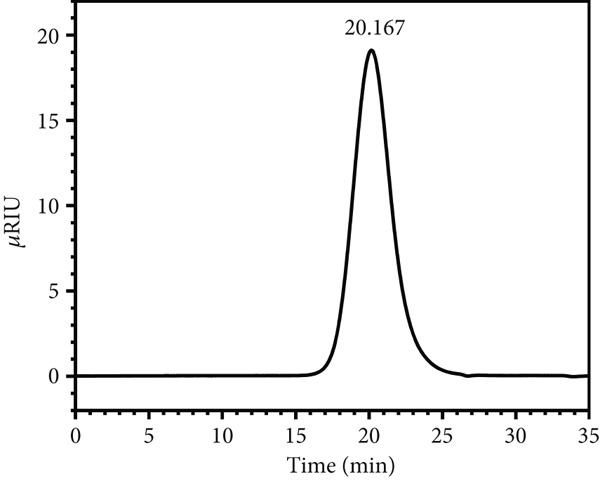
(a)

**Figure:**
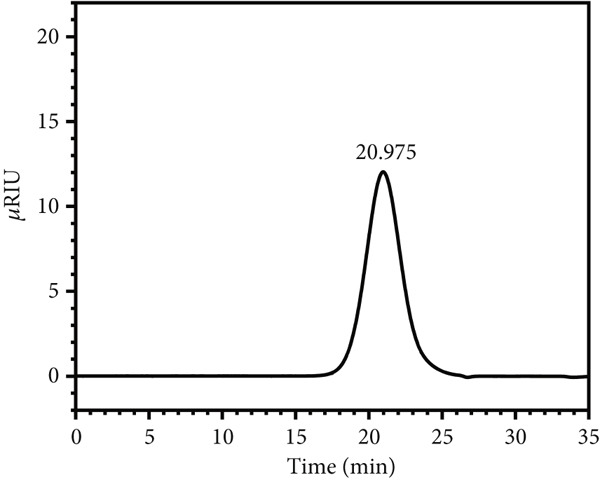
(b)

**Figure:**
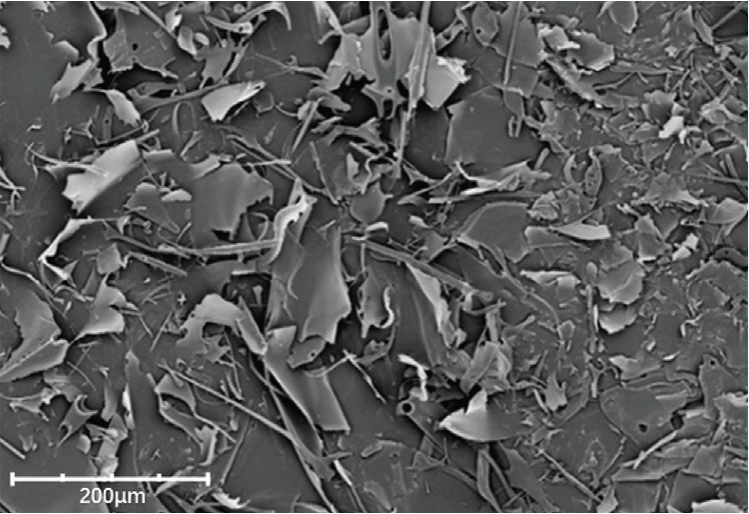
(c)

**Figure:**
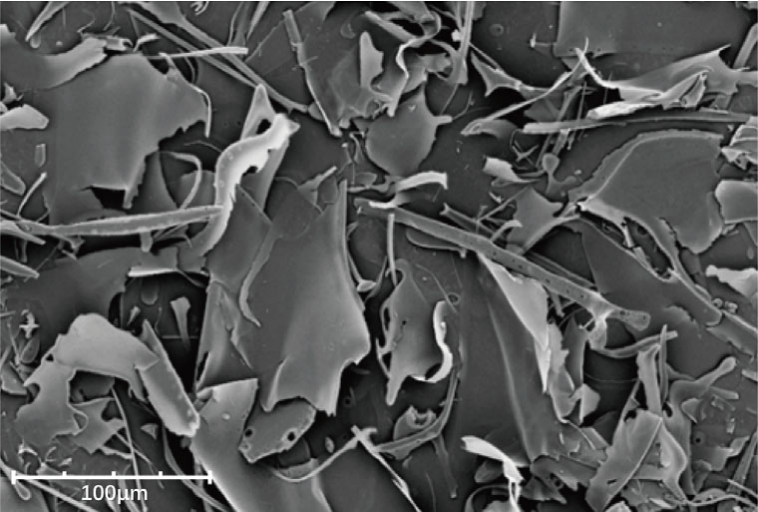
(d)

**Figure:**
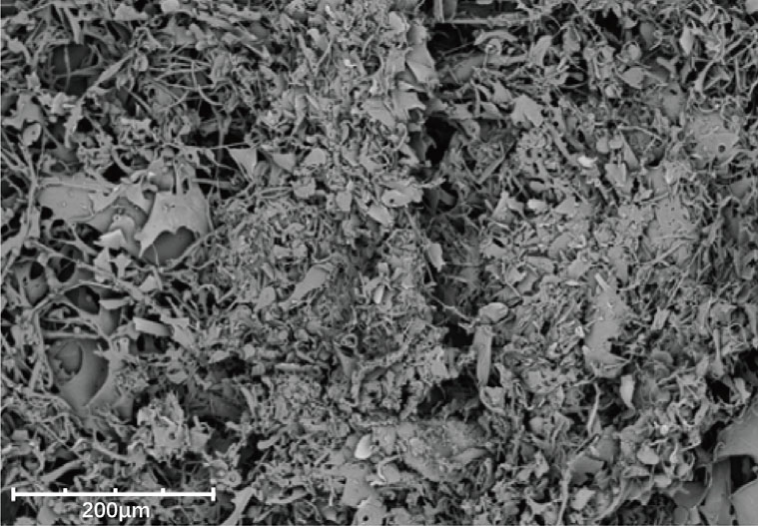
(e)

**Figure:**
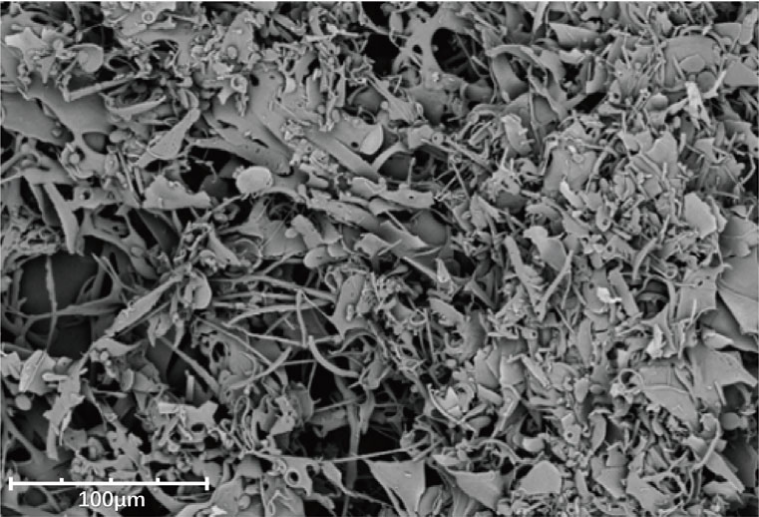
(f)

### 3.7. Scanning Electron Microscopy (SEM) Analysis

SEM provided further insights into their morphological characteristics (Figures 5c, 5d, 5e, and 5f). SEM images revealed notable differences: LBP‐1 appeared as the aggregated, sheet‐like structures with smooth, compact surfaces, whereas LBP‐2 exhibited a seminetwork, fragmented morphology with more dispersed, fibrous features. These structural differences likely related to their molecular weights, with the higher molecular weight of LBP‐1 contributing to a denser, cohesive structure and the lower molecular weight of LBP‐2 resulting in a looser, fragmented morphology. This correlation between molecular weight and morphology supported the HPGPC findings, where LBP‐1 formed more compact structures and LBP‐2 formed more dispersed ones.

### 3.8. XRD Analysis

XRD analysis (Figure 6a,b) was used to evaluate the molecular ordering of LBP subfractions. LBP‐1 exhibited sharp peaks, particularly at 28.2° and 40.5°, indicating the presence of locally ordered or microcrystalline regions. A few minor sharp peaks were also observed at higher angles, which may partially arise from residual inorganic salts introduced during extraction or chromatography. In contrast, LBP‐2 displayed broad, low‐intensity reflections centered around 13.4° and 29.7°, consistent with a predominantly amorphous structure. These observations are supported by SEM imaging, which revealed that LBP‐1 tends to form compact sheet‐like assemblies with smooth surfaces, whereas LBP‐2 adopts a more dispersed and irregular morphology with fragmented, fibrous textures. The combined evidence suggests that LBP‐1 possesses tighter molecular packing and higher local order, while LBP‐2 exists in a less organized, loosely associated state. Such variations in structural arrangement underscore the inherent heterogeneity of polysaccharide assemblies.

Figure 6Morphological and structural analysis of LBP‐1 and LBP‐2. (a) XRD pattern of LBP‐1. (b) XRD pattern of LBP‐2. (c) Congo red analysis of triple‐helix structure in LBP‐1 and LBP‐2. (d) Ion chromatogram of monosaccharide standards. (e) Ion chromatogram of LBP‐1. (f) Ion chromatogram of LBP‐2.

**Figure:**
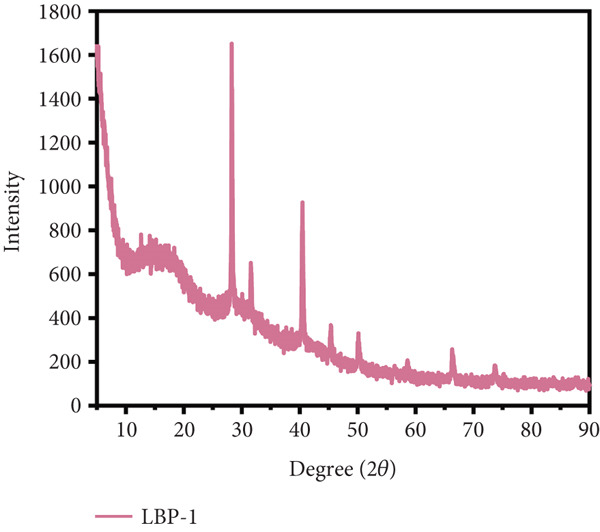
(a)

**Figure:**
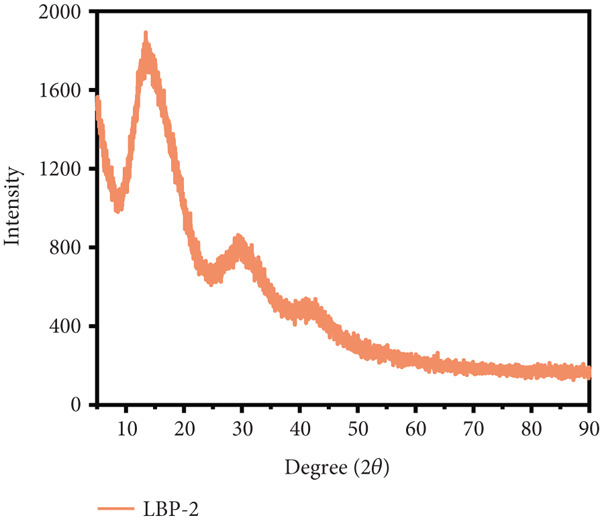
(b)

**Figure:**
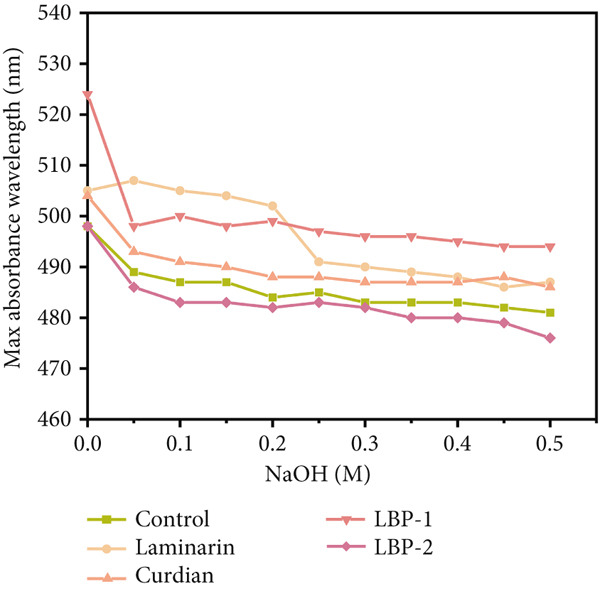
(c)

**Figure:**
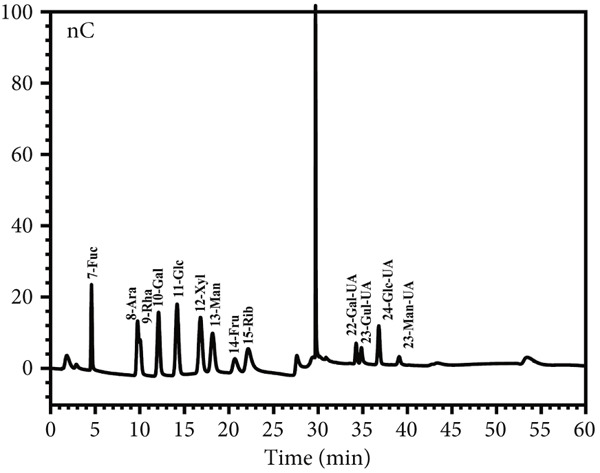
(d)

**Figure:**
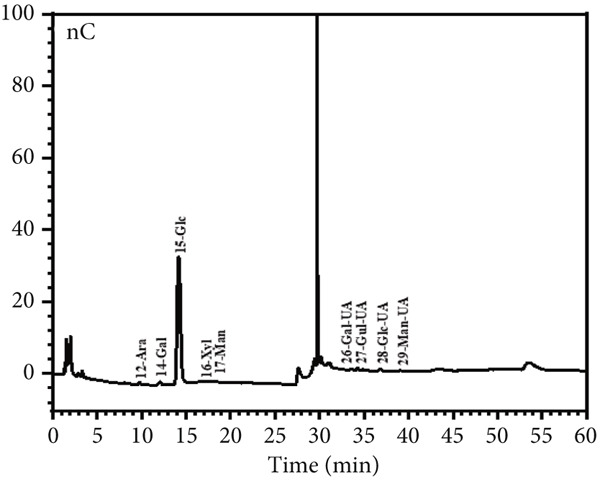
(e)

**Figure:**
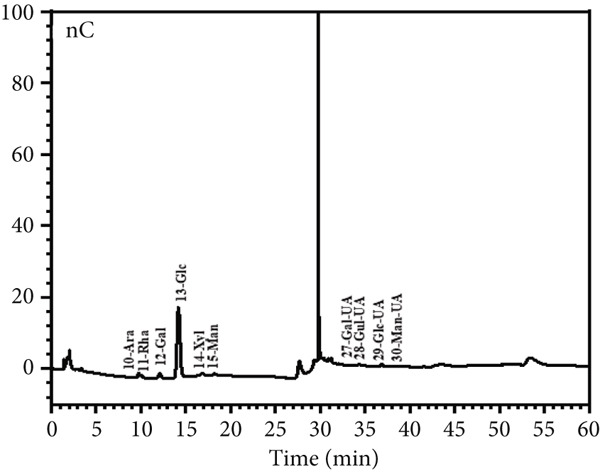
(f)

### 3.9. Triple‐Helix Structure and Monosaccharide Composition Analysis of LBP Subfractions

The triple‐helix structures of LBP‐1 and LBP‐2 were investigated utilizing the Congo red assay, with the findings presented in Figure 6c. The interaction of LBP‐1 with Congo red resulted in a red shift of the maximum absorption wavelength from 498 to 524 nm, thereby confirming the existence of a triple‐helix conformation. In contrast, LBP‐2 showed no significant red shift compared to the blank control, suggesting that it lacks a triple‐helix structure.

The observed conformational variations may be attributed to the molecular weight and glycosidic bond types characteristic of each subfraction. Specifically, LBP‐1, which possessed a higher molecular weight and contained both *α*‐ and *β*‐pyranose linkages, was likely to adopt a more intricate, helical conformation. In contrast, LBP‐2, characterized by a lower molecular weight and exclusively *β*‐pyranose linkages, appeared to lack such structural complexity. IC was used to analyze the monosaccharide composition of LBP‐1 and LBP‐2. The chromatogram for the standard mixture of 13 monosaccharides (Figure 6d) showed distinct, well‐resolved peaks. Using calibration curves from these standards, the monosaccharide compositions of LBP‐1 and LBP‐2 were identified and quantified by retention times and peak areas (Figure 6e,f). In LBP‐1 (Figure 6e), the primary monosaccharides included Ara, Gal, Glc, Xyl, Man, and several uronic acids such as Gal‐UA, Gul‐UA, Glc‐UA, and Man‐UA. Among these, Glc, Man‐UA, GalUA, and Glc‐UA were the most abundant, indicating a relatively high content of both neutral and acidic monosaccharides, particularly uronic acids, in LBP‐1. LBP‐2 (Figure 6f) showed a similar monosaccharide profile but with elevated levels of Ara, Rha, and Man, along with higher uronic acid content, particularly Gal‐UA and Man‐UA. Such differences in sugar composition, especially the enrichment of uronic acids and Rha, may contribute to LBP‐2′s distinct physicochemical behavior and potential functional attributes. These features could affect solubility, molecular interactions, or conformational flexibility, which are often linked to biological activity in polysaccharides [38, 39]. A detailed comparison of the monosaccharide composition between LBP‐1 and LBP‐2 is provided in Table 4. Compared with LBP‐1, LBP‐2 contained a higher proportion of uronic acids, such as Gal‐UA and Man‐UA, along with increased levels of Ara and Rha. These compositional differences may reflect variations in sugar branching or structural organization between the two subfractions.

**Table 4:** Monosaccharide composition of LBP‐1 and LBP‐2.

**Name**	**LBP-1 component content (*μ*g/mg)**	**Proportion of each LBP-1 component (%)**	**LBP-2 component content (*μ*g/mg)**	**Proportion of each LBP-2 component (%)**
Ara	2.7769	1.68	6.4202	5.14
Rha	0.0000	0.00	4.1084	3.29
Gal	3.8199	2.30	6.1711	4.94
Glc	131.6246	79.40	76.9829	61.58
Xyl	0.8580	0.52	4.2901	3.43
Man	1.3719	0.83	3.8066	3.04
Gal‐UA	8.2417	4.97	8.6512	6.92
Gul‐UA	2.6909	1.62	1.7899	1.43
Glc‐UA	4.7086	2.84	4.9781	3.98
Man‐UA	9.6840	5.84	7.8187	6.25

### 3.10. Thermal Properties

As shown in Figure 7a, the DSC thermograms of LBP‐1 and LBP‐2 displayed markedly different endothermic profiles. LBP‐1 exhibited two distinct endothermic transitions at 130.25°C and 147.38°C, indicating a relatively high thermal resistance. These thermal events suggest the presence of ordered molecular domains, likely stabilized by interchain hydrogen bonding or triple‐helix conformations. Such structures can hinder polymer chain mobility, resulting in elevated glass transition temperatures. In contrast, LBP‐2 showed a single, broad endothermic peak centered at 97.47°C. The lower transition point reflects reduced thermal stability and implies a more flexible, amorphous molecular arrangement. The absence of higher temperature transitions suggests limited formation of ordered or helical structures in this fraction.

Figure 7(a) Differential scanning calorimetry (DSC) thermograms of LBP‐1 and LBP‐2. (b) ABTS^+^ radical scavenging capacity of LBP‐1, LBP‐2, and controls. (c) DPPH radical scavenging capacity of LBP‐1, LBP‐2, and controls. (d) Hydroxyl radical scavenging capacity of LBP‐1, LBP‐2, and controls.

**Figure:**
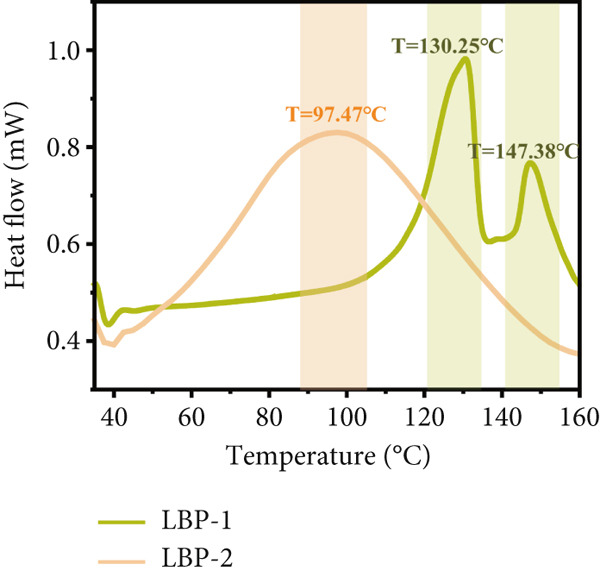
(a)

**Figure:**
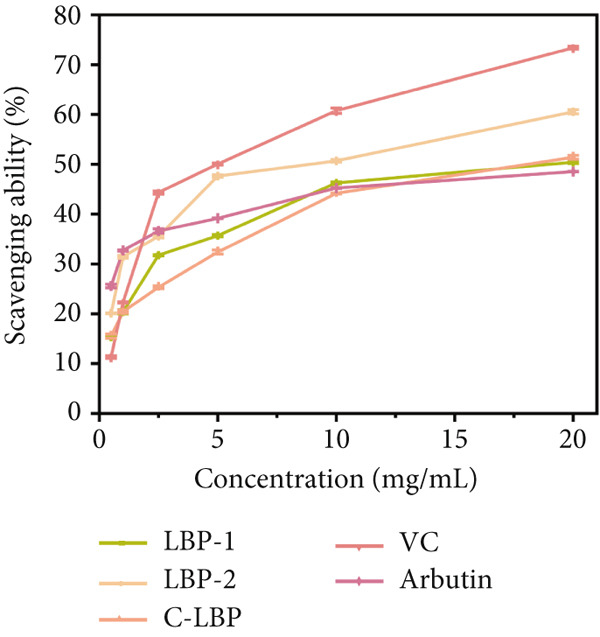
(b)

**Figure:**
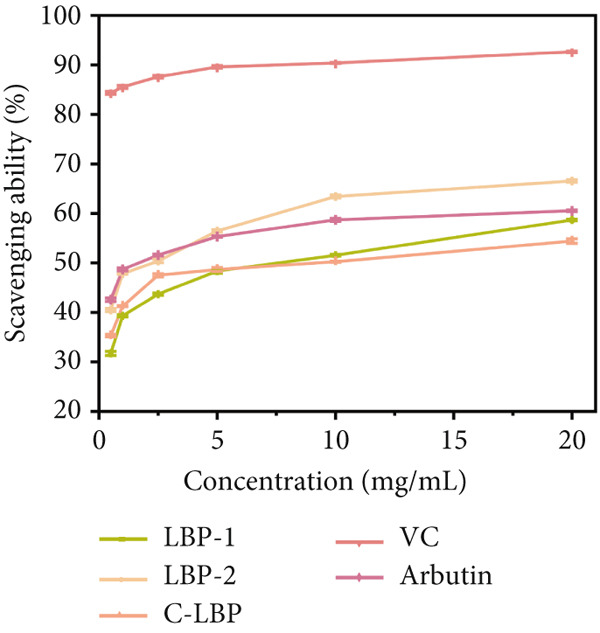
(c)

**Figure:**
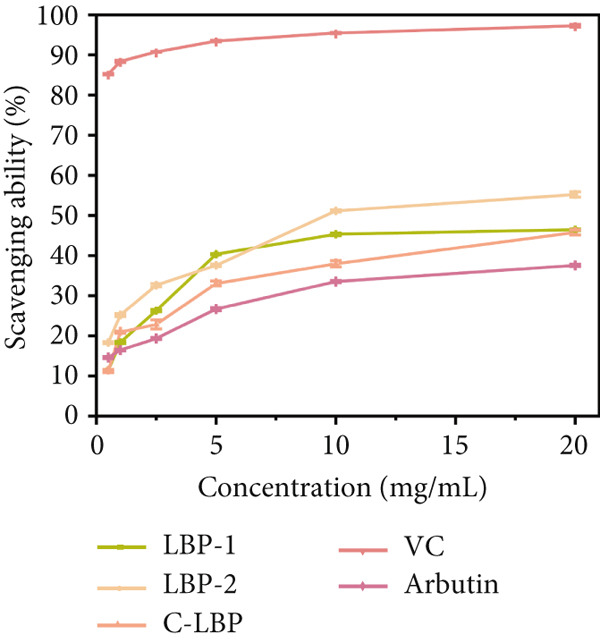
(d)

These distinct thermal behaviors indicate that LBP‐1 is characterized by tighter molecular packing and greater structural order, while LBP‐2 exhibits a more disordered and thermally labile conformation.

### 3.11. Antioxidant Activity of Purified LBP Subfractions

The antioxidant activities of LBP‐1 and LBP‐2 were assessed using ABTS^+^, DPPH, and hydroxyl radical scavenging assays, with crude LBP, vitamin C (VC), and arbutin serving as controls.

In the ABTS^+^ scavenging assay (Figure 7b), ABTS^+^ forms a stable blue–green radical cation in methanol, characterized by an absorption peak at 744 nm [28]. Antioxidants can eliminate these radicals, leading to a reduction in color intensity. Scavenging capacities were measured at various concentrations for LBP‐1, LBP‐2, crude LBP, VC, and arbutin. The results demonstrated a concentration‐dependent enhancement in scavenging activity across all samples. VC exhibited the highest scavenging capacity, achieving a 73.39% removal rate at a concentration of 20 mg/mL. LBP‐2 also demonstrated significant activity, with a 60.54% removal rate at the same concentration, surpassing the performance of LBP‐1, crude LBP, and arbutin. The enhanced performance of LBP‐2 may be related to its relatively loose and fragmented structure, which could expose more functional groups such as hydroxyl and carboxyl moieties. This increased exposure may provide more accessible active sites for radical scavenging. In contrast, the lower activity of LBP‐1 and crude LBP may be attributed to the lower polysaccharide content in the crude sample and the more compact structure of LBP‐1, which may limit the exposure of reactive groups.

In the DPPH radical scavenging assay (Figure 7c), DPPH in ethanol forms a stable purple compound with an absorption peak at 517 nm [29]. Antioxidants can eliminate these radicals, causing the color to fade. Both LBP‐1 and LBP‐2 exhibited a concentration‐dependent increase in DPPH scavenging activity across the 0.5 ~ 20 mg/mL range, with VC showing the strongest activity, achieving a 92.66% scavenging rate at 20 mg/mL concentration. LBP‐2 exhibited significant DPPH radical scavenging activity, reaching 66.55% at the same concentration, surpassing LBP‐1, crude LBP, and arbutin. Its enhanced performance may stem from a more accessible structure that facilitates interaction with free radicals.

Similarly, the hydroxyl radical scavenging activity of LBP‐1 and LBP‐2 was evaluated (Figure 7d). Hydroxyl radicals are highly reactive and could damage biological macromolecules, contributing to cell death and various diseases. This assay measured scavenging activity by observing the reaction between hydroxyl radicals and salicylic acid, which formed colored compounds with an absorption peak at 510 nm [40]. LBP‐1, LBP‐2, and crude LBP all showed hydroxyl radical scavenging activity, increasing with concentration across the 0.5 ~ 20 mg/mL range. Once again, VC showed the highest efficiency, reaching 97.28% at 20 mg/mL concentration. LBP‐2 achieved a 55.23% hydroxyl radical scavenging rate, markedly surpassing the performance of LBP‐1, crude LBP, and arbutin. This superior activity was likely attributed to its structural features, such as higher uronic acid content and greater exposure of reactive groups, which enhanced its ability to neutralize highly reactive hydroxyl radicals. In contrast, the lower activity of LBP‐1 was possibly due to its tighter molecular packing, which limited the accessibility of functional sites. These results highlight the potential of LBP‐2 as a natural antioxidant candidate for food preservation and formulation of health‐promoting food products.

### 3.12. Inhibition of Tyrosinase Activity by Purified LBP Subfractions

The inhibitory effects of LBP‐1 and LBP‐2 on tyrosinase activity were assessed using the L‐DOPA oxidation assay, which measured the conversion of L‐DOPA to dopachrome, a precursor in melanin synthesis [41]. The time‐dependent effects (0 ~ 20 min) and concentration‐dependent inhibitory effects of LBP‐1 and LBP‐2 are shown in Figure 8a,b. As illustrated in Figure 8a, both LBP‐1 and LBP‐2 inhibited tyrosinase activity at all tested concentrations, as indicated by lower absorbance at 475 nm (reflecting dopachrome formation) compared to the control. Increasing concentrations of LBP‐1 and LBP‐2 led to further reductions in absorbance, demonstrating concentration‐dependent inhibition. Notably, LBP‐2 showed a stronger inhibitory effect than LBP‐1 at equivalent concentrations, as evidenced by consistently lower absorbance values. Figure 8b further illustrated the dose‐dependent inhibition of tyrosinase by LBP‐1 and LBP‐2. LBP‐2 consistently exhibited higher inhibitory rates than LBP‐1, with IC_50_ values calculated as 138.0 *μ*g/mL for LBP‐2 and 56.2 *μ*g/mL for LBP‐1, based on their respective weight‐average molecular weights determined by gel filtration. These results confirmed the significantly stronger tyrosinase inhibition by LBP‐2. At higher concentrations (1.0 ~ 2.0 mg/mL), LBP‐2′s inhibition rate approached that of arbutin, a known tyrosinase inhibitor, suggesting its potential as an effective natural alternative.

Figure 8(a) Time‐course inhibition of tyrosinase activity by LBP‐1 and LBP‐2 (0 ~ 20 min). (b) Inhibition rates of LBP‐1 and LBP‐2 at varying concentrations. (c) Lineweaver–Burk plot of LBP‐1. (d) Lineweaver–Burk plot of LBP‐2. (e–h) Molecular docking of representative monosaccharide residues with tyrosinase: (e) arabinose (Ara), (f) galacturonic acid (Gal‐UA), (g) glucose (Glc), and (h) rhamnose (Rha).

**Figure:**
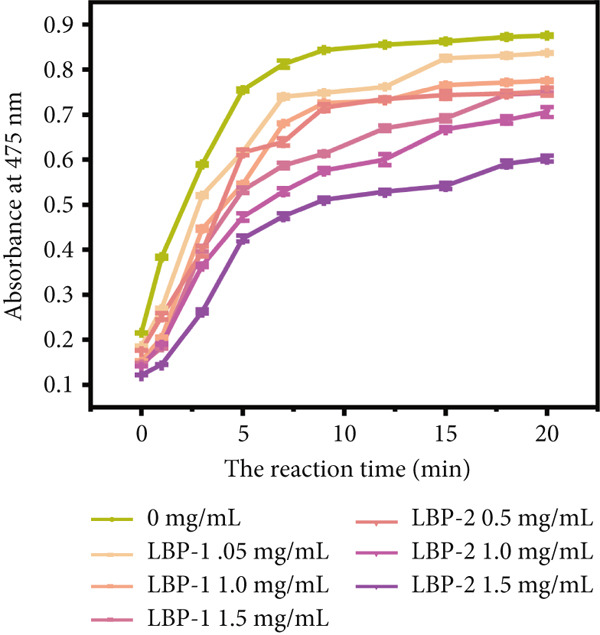
(a)

**Figure:**
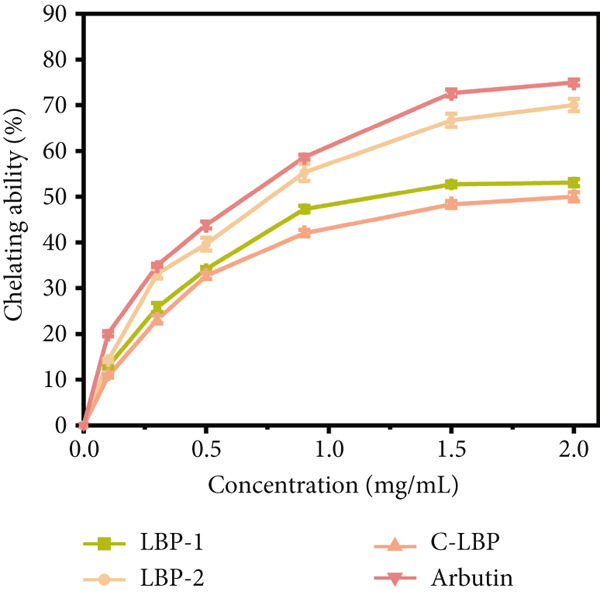
(b)

**Figure:**
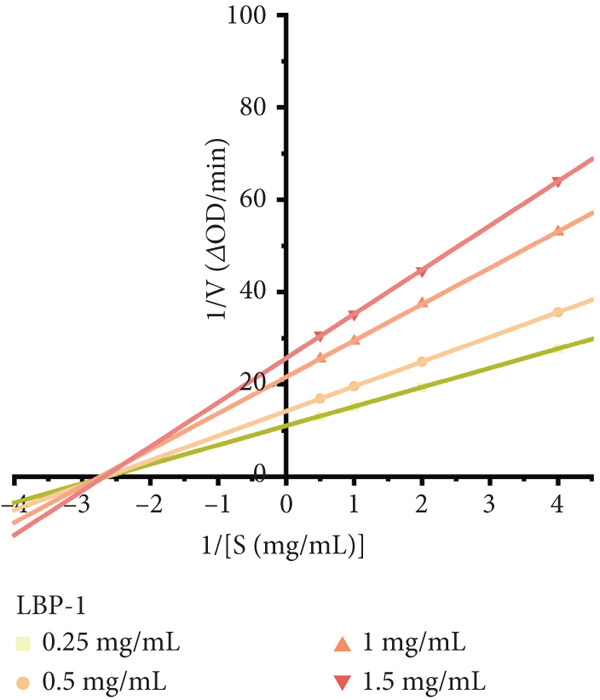
(c)

**Figure:**
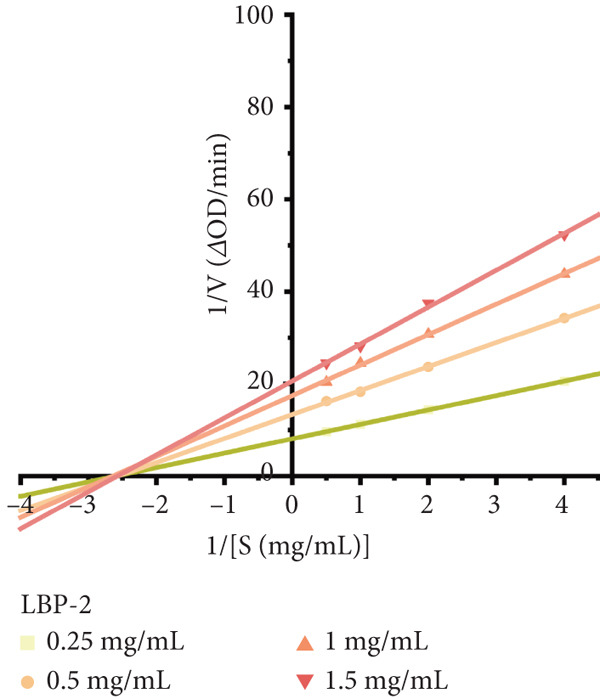
(d)

**Figure:**
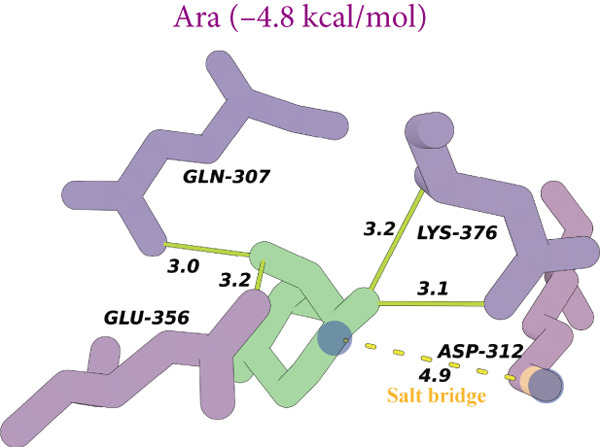
(e)

**Figure:**
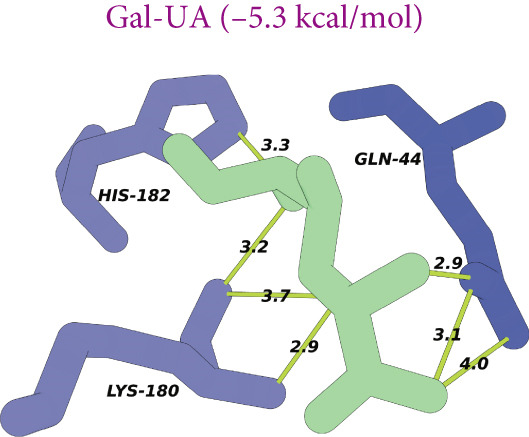
(f)

**Figure:**
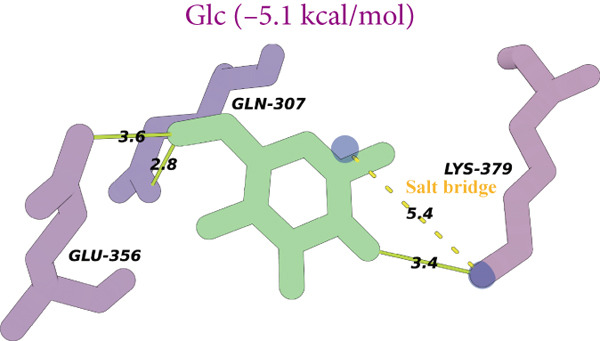
(g)

**Figure:**
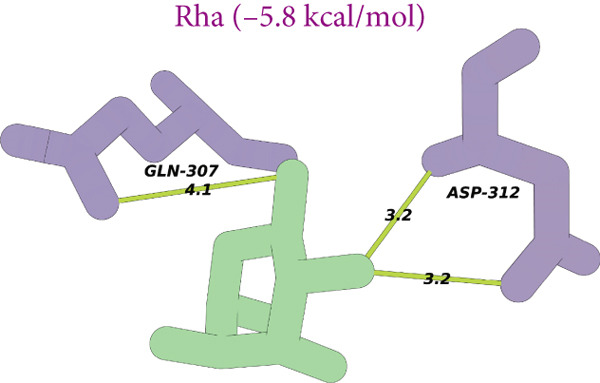
(h)

To determine the type of inhibition exerted by LBP‐1 and LBP‐2 on tyrosinase, Lineweaver–Burk double‐reciprocal plots were generated (Figure 8c,d). Kinetic analysis indicated that both subfractions acted as noncompetitive reversible inhibitors. As the concentration of LBP‐1 and LBP‐2 increased from 0.25 to 1.5 mg/mL, the *x*‐axis intercepts (1/[*S*], inverse substrate concentration) remained unchanged, suggesting no effect on substrate binding affinity (*K*_m_) [42]. However, the *y*‐axis intercepts (1/*V*_max_, inverse maximum reaction velocity) shifted, indicating a reduction in the maximum catalytic rate (*V*_max_) of tyrosinase without affecting substrate binding—characteristic of noncompetitive inhibition. The kinetic parameters (*K*_m_ and *V*_max_) calculated from the Lineweaver–Burk plots were summarized in Tables 5 and 6, confirming that LBP‐1 and LBP‐2 acted as noncompetitive inhibitors. This indicates that they bind to tyrosinase at a site other than the active site, reducing its catalytic efficiency without directly competing with L‐DOPA.

**Table 5:** Kinetic parameters of tyrosinase inhibition by LBP‐1.

**Concentration of LBP-1 (mg/mL)**	**Lineweaver–Burk equation**	**K**_**m**_	**V**_**m**_
0.25	1/*V* = 1/[*S*] 4.173 + 11.026	0.378	0.091
0.5	1/*V* = 1/[*S*] 5.351 + 14.206	0.377	0.070
1.0	1/*V* = 1/[*S*] 7.880 + 21.592	0.365	0.046
1.5	1/*V* = 1/[*S*] 9.581 + 25.661	0.373	0.039

**Table 6:** Kinetic parameters of tyrosinase inhibition by LBP‐2.

**Concentration of LBP-2 (mg/mL)**	**Lineweaver–Burk equation**	**K**_**m**_	**V**_**m**_
0.25	1/*V* = 1/[*S*] 3.119 + 8.106	0.384	0.123
0.5	1/*V* = 1/[*S*] 5.203 + 13.357	0.390	0.075
1.0	1/*V* = 1/[*S*] 6.603 + 17.478	0.378	0.057
1.5	1/*V* = 1/[*S*] 8.003 + 20.619	0.388	0.049

In conclusion, both LBP‐1 and LBP‐2 exhibited notable noncompetitive inhibitory effects on tyrosinase, with LBP‐2 showing stronger inhibition across all tested concentrations. This suggests that LBP‐2 may serve as a promising natural tyrosinase inhibitor in food systems, especially in applications aimed at preventing enzymatic browning or mitigating oxidative deterioration in fruits, beverages, and functional food formulations.

### 3.13. Molecular Docking Analysis

As shown in Figures 8e, 8f, 8g, and 8h, the four tested monosaccharides (Ara, GalUA, Glc, and Rha) exhibited stable binding to the active site of tyrosinase (PDB ID: 2Y9X), with predicted binding energies ranging from 4.8 to 5.8 kcal/mol. Rha displayed the strongest affinity, followed by GalUA, Glc, and Ara. Ara and Glc formed salt bridges with the catalytic copper ions, which may interfere with substrate access or disrupt the oxidative activity of the active site. In contrast, GalUA and Rha formed hydrogen bonds with surrounding residues but did not directly coordinate with the copper center.

These findings offer a mechanistic explanation for the stronger tyrosinase‐inhibitory effect observed in LBP‐2, which contains higher proportions of Ara and GalUA compared to LBP‐1. The ability of these sugars to engage the enzyme′s catalytic core may contribute to the enhanced bioactivity of LBP‐2. While simplified, this docking analysis provides preliminary insight into how specific monosaccharides influence inhibitory performance.

Future studies may further refine the structure–function relationship by exploring how glycosidic linkages and uronic acid content modulate bioactivity. Cell‐based assays, such as B16 melanogenesis models, could also verify whether LBP‐2 suppresses melanin production without inducing cytotoxicity [43]. From an application perspective, LBP‐2 shows promise as a clean‐label antioxidant and antibrowning agent for use in cut fruits, herbal infusions, and plant‐based beverages. Improving extraction and purification efficiency could enhance its functional yield and commercial potential [9, 44, 45]. Further optimization of delivery forms or coformulation with food‐grade stabilizers may also improve its solubility, stability, and efficacy in complex food matrices.

## 4. Conclusion

In this study, the optimal conditions for hot water extraction of LBPs were determined to be a liquid‐to‐solid ratio of 30 mL/g, an extraction temperature of 90°C, and an extraction time of 3 h, resulting in a polysaccharide yield of 7.44*%* ± 0.17*%*. Two major fractions, LBP‐1 and LBP‐2, were purified through DEAE‐52 ion‐exchange and Sephadex G‐150 gel‐filtration chromatography, with molecular weights of 115.1 and 73.5 kDa, respectively.

Structural analysis revealed that LBP‐1 possessed a relatively compact and possibly subcrystalline surface morphology, exhibited a triple‐helix conformation, and contained both *α*‐ and *β*‐glycosidic linkages. Its major monosaccharide components included Glc, Man‐UA, GalUA, and Glc‐UA. In contrast, LBP‐2 displayed a more fragmented and amorphous structure without triple‐helix characteristics, featured predominantly *β*‐linkages, and was rich in Glc, GalUA, Man‐UA, Ara, and Rha. Both LBP‐1 and LBP‐2 exhibited notable antioxidant and tyrosinase‐inhibitory activities. LBP‐1 may benefit from its ordered conformation and acidic sugar content, while LBP‐2 showed stronger overall activity, likely due to its more open structure, higher Ara and uronic acid content, and the presence of sugar residues capable of targeting the catalytic center of tyrosinase, as supported by molecular docking results. Overall, the structural characterization and functional evaluation of these well‐defined LBP subfractions provide valuable insights into the composition–activity relationship of LBPs and support their potential application as natural, clean‐label bioactives in functional foods and antioxidant formulations.

## Conflicts of Interest

The authors declare no conflicts of interest.

## Author Contributions

**Yuling Chen:** software, data curation, methodology, writing—original draft; **Xueluan Liu:** software, data curation, methodology, writing—original draft; **Genglin Lu:** conceptualization, investigation, software, methodology, data curation, formal analysis, and validation; **Xuan He:** data curation, formal analysis, and validation; **Dandan Li:** data curation, visualization, and validation; **Yunong Tian:** data curation, resources, and validation; **Xiaolin Feng:** data curation, formal analysis, and validation; **Yong Ye:** resources, investigation, formal analysis, supervision, writing—original draft, and project administration. **Yuling Chen** and **Xueluan Liu** contributed equally.

## Funding

This study was supported by the Science and Technology Plan Project of Guangzhou City, 2024B03J1269; Qingyuan City Science and Technology Plan Projects, 2021DZX016, 2022DZX009, and 2023DZX007; and Jiangxi Provincial Forestry Bureau Camellia Oleifera Research Special Project, YCYJZX[2023]311.

## Supporting information


**Supporting Information** Additional supporting information can be found online in the Supporting Information section. To assess the adequacy and reliability of the response surface methodology (RSM) model, diagnostic plots and statistical indices were analyzed, as shown in Figure S2. SDS‐PAGE analysis of purified LBP is shown in Figure S2. Chemical shifts in resonances in the NMR spectra of LBP‐1 and LBP‐2 are listed in Table S1 and Table S2, respectively. Figure S1: Diagnostic plots for model validation. (a) Box–Cox plot of Ln(residual SS) versus *λ*, indicating no transformation needed. (b) Normal probability plot showing that residuals follow a normal distribution. (c) Actual versus predicted values showing strong model fit. (d) Residuals versus predicted values indicating constant variance. Figure S2: SDS‐PAGE analysis of purified LBP. Table S1: Chemical shifts in resonances in the NMR spectra of LBP‐1. Table S2: Chemical shifts in resonances in the NMR spectra of LBP‐2.

## Data Availability

The data in this research are available on request.
