# Supplementary material for: Properties of Novel Components of Polysaccharides Isolated From Lycium barbarum Fruits
Source: Int J Food Sci. 2025 Nov 28;2025:4899439. doi: 10.1155/ijfo/4899439 (PMC12662143; doi:10.1155/ijfo/4899439)
Supplement: Supplementary file 1 — Supporting Information Additional supporting information can be found online in the Supporting Information section. To assess the adequacy and reliability of the response surface methodology (RSM) model, diagnostic plots and statistical indices were analyzed, as shown in Figure S2. SDS‐PAGE analysis of purified LBP is shown in Figure S2. Chemical shifts in resonances in the NMR spectra of LBP‐1 and LBP‐2 are listed in Table S1 and Table S2, respectively. Figure S1: Diagnostic plots for model validation. (a) Box–Cox plot of Ln(residual SS) versus λ, indicating no transformation needed. (b) Normal probability plot showing that residuals follow a normal distribution. (c) Actual versus predicted values showing strong model fit. (d) Residuals versus predicted values indicating constant variance. Figure S2: SDS‐PAGE analysis of purified LBP. Table S1: Chemical shifts in resonances in the NMR spectra of LBP‐1. Table S2: Chemical shifts in resonances in the NMR spectra of LBP‐2. [file IJFO-2025-4899439-s001.docx]

Properties of novel components of polysaccharides isolated from *Lycium barbarum* fruits

Yuling Chen^a,#^, Xueluan Liu^a,#^, Genglin Lu^a^, Xuan He^b^, Dandan Li^c^, Yunong Tian^a^, Xiaolin Feng^a^, Ye Yong^a,c*^

^a^ *Department of Pharmaceutical Engineering, School of Chemistry and Chemical Engineering, South China University of Technology, Guangzhou, 510640, China*

^b^ *Department of R&D, Ganzhou Hake Biotech Co., Ltd, Ganzhou, 341008, China*

*^c^ Department of Forestry, Jiangxi Environmental Engineering Vocational College, Ganzhou, 341000, China*

^#^ *These authors contributed equally*

^*^ Corresponding authors:

E-mail: yeyong@scut.edu.cn (Y. Ye)

Phone and Fax: +86-20-871102

**Part S1. Diagnostic Evaluation of the RSM Model**

To assess the adequacy and reliability of the response surface methodology (RSM) model, diagnostic plots and statistical indices were analyzed, as shown in Figure S1. The Box–Cox plot (Figure S1a) displays the natural logarithm of the residual sum of squares (Ln(residual SS)) versus the transformation parameter lambda (λ). The curve reaches its minimum near λ = 1, indicating that no data transformation is necessary and that the original model form is appropriate. The normal probability plot of externally studentized residuals (Figure S1b) shows that the residuals are closely aligned along the diagonal reference line. This pattern suggests that the residuals approximate a normal distribution, fulfilling one of the key assumptions for regression-based modeling.

The scatter plot of actual versus predicted extraction yields (Figure S1c) demonstrates that most points fall near the 45-degree reference line, indicating good agreement between the experimental and model-predicted values. Meanwhile, the plot of externally studentized residuals versus predicted values (Figure S1d) shows no discernible structure or trend, supporting the assumption of constant variance (homoscedasticity). In addition to visual diagnostics, the model’s goodness of fit was evaluated numerically. The coefficient of determination (R^2^) was 0.9867, the adjusted R^2^ was 0.9721, and the predicted R^2^ was 0.9264. These values reflect high explanatory power, minimal overfitting, and strong predictive ability, collectively confirming that the fitted model is statistically sound and suitable for process optimization.


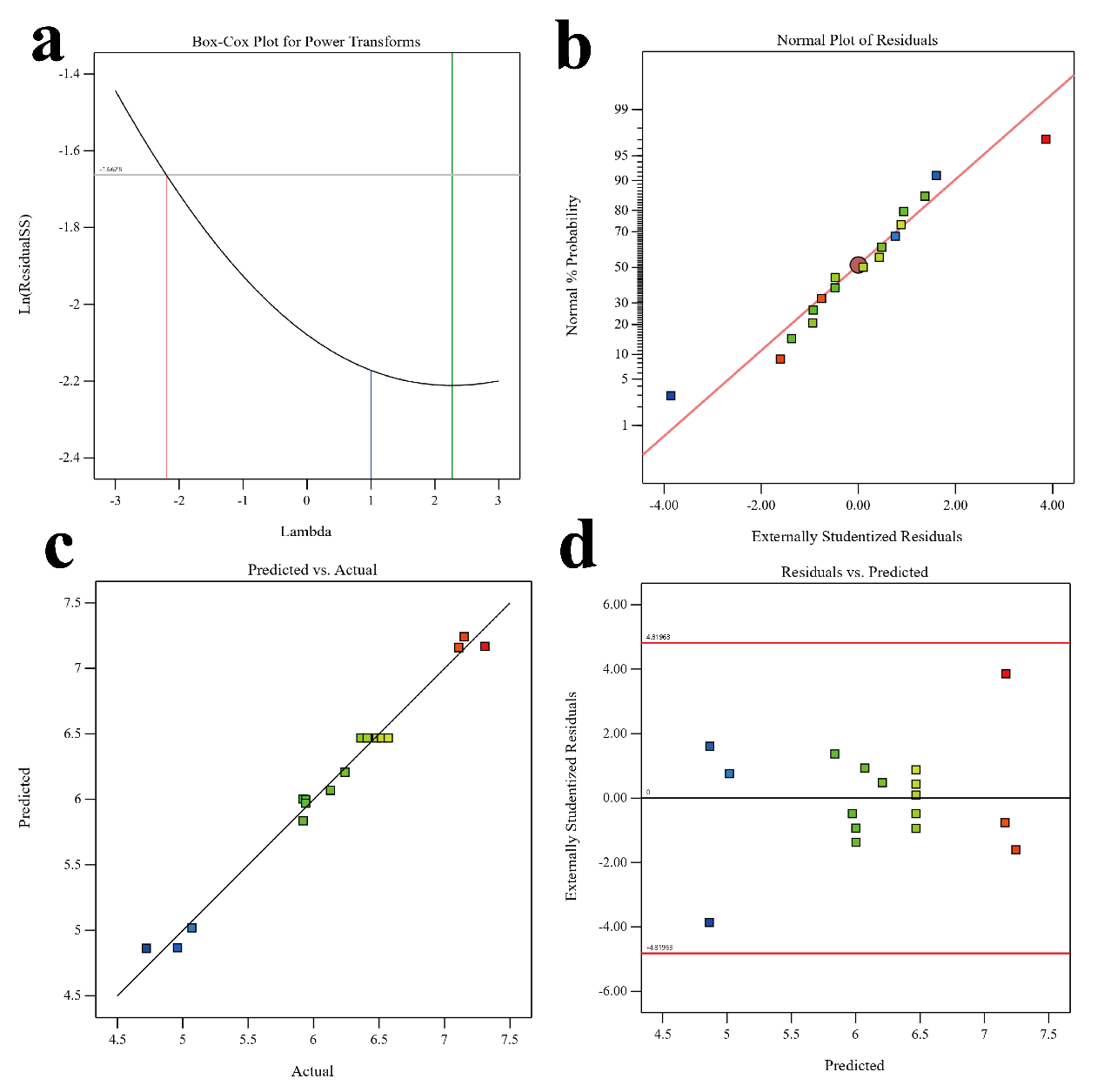


Figure S1. Diagnostic plots for model validation. (a) Box–Cox plot of Ln(residual SS) versus λ, indicating no transformation needed. (b) Normal probability plot showing that residuals follow a normal distribution. (c) Actual versus predicted values showing strong model fit. (d) Residuals versus predicted values indicating constant variance.


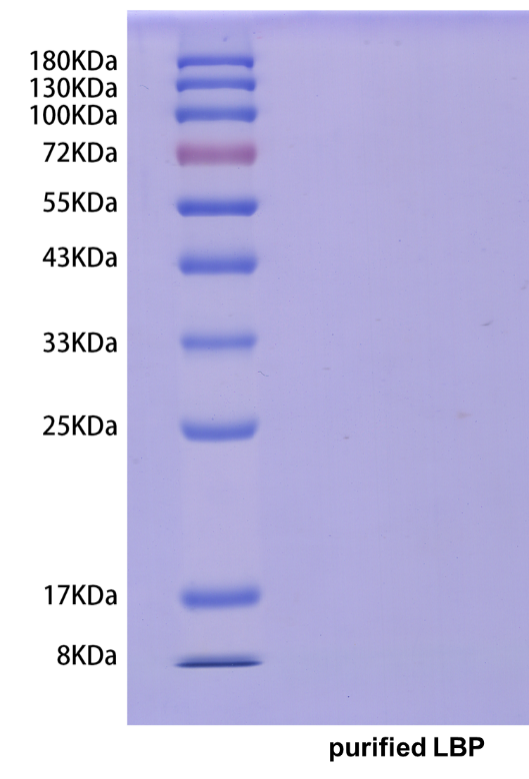


Figure S2. SDS-PAGE analysis of purified LBP.

**Table S1.** Chemical shifts in resonances in the NMR spectra of LBP-1.

| Glycosyl Residues |  | Chemical Shifts (ppm) | | | | |
| --- | --- | --- | --- | --- | --- | --- |
|  |  | 1 | 2 | 3 | 4 | 5 |
| →3,4)-α-Galp-(1→ | H | 5.20 | 4.13 | 3.82 | 3.69 | 3.25 |
|  | C | 101.49 | 80.67 | 74.14 | 69.25 | 63.89 |
| →3)-α-Galp-(1→ | H | 5.26 | 4.06 | 3.72 | 3.60 | 3.27 |
|  | C | 98.07 | 75.41 | 73.84 | 70.69 | 62.92 |
| →4)-β-Arap-(1→ | H | 4.79 | 4.02 | 3.68 | 3.51 | 3.22 |
|  | C | 95.90 | 75.75 | 70.92 | 67.89 | 60.75 |
| β-Arap-(1→ | H | 4.67 | 4.03 | 3.66 | 3.44 | 3.19 |
|  | C | 94.03 | 73.84 | 71.47 | 67.56 | 60.60 |
| →3,4)-β-Arap-(1→ | H | 4.66 | 4.02 | 3.60 | 3.42 | 2.81 |
|  | C | 92.09 | 75.94 | 72.07 | 69.67 | 61.79 |
| →3)-α-Glc*p*-(1→ | H | 5.25 | 4.14 | 3.80 | 3.65 | 3.28 |
|  | C | 95.90 | 77.22 | 73.84 | 69.42 | 63.37 |

**Table S2.** Chemical shifts in resonances in the NMR spectra of LBP-2.

| Glycosyl Residues |  | Chemical Shifts (ppm) | | | | |
| --- | --- | --- | --- | --- | --- | --- |
|  |  | 1 | 2 | 3 | 4 | 5 |
| →4)-β-Gal*p*-(1→ | H | 4.67 | 3.91 | 3.78 | 3.63 | 3.41 |
|  | C | 99.74 | 76.75 | 74.53 | 71.29 | 69.18 |
| α-Glc*p*-(1→ | H | 5.42 | 3.97 | 3.76 | 3.55 | 3.30 |
|  | C | 98.05 | 72.86 | 71.39 | 69.31 | 60.41 |
| →3)-α-Glc*p*-(1→ | H | 5.24 | 4.05 | 3.86 | 3.58 | 3.23 |
|  | C | 95.90 | 76.17 | 74.12 | 71.16 | 63.88 |
| β-Glc*p*-(1→ | H | 4.99 | 4.12 | 3.91 | 3.67 | 3.25 |
|  | C | 97.70 | 75.93 | 73.99 | 69.59 | 60.74 |
| →3,4)-β-Ara*p*-(1→ | H | 4.66 | 3.92 | 3.75 | 3.53 | 3.29 |
|  | C | 99.59 | 74.12 | 71.53 | 69.67 | 65.53 |
| →3)-α-Ara*p*-(1→ | H | 5.00 | 4.11 | 3.73 | 3.49 | 3.27 |
|  | C | 95.77 | 75.75 | 73.32 | 71.72 | 63.35 |
| →4)-α-Glc*p*-(1→ | H | 5.23 | 3.84 | 3.65 | 3.43 | 2.12 |
|  | C | 91.89 | 76.75 | 72.70 | 70.17 | 60.47 |
